# Mutant generation by allelic exchange and genome resequencing of the biobutanol organism *Clostridium acetobutylicum* ATCC 824

**DOI:** 10.1186/s13068-015-0410-0

**Published:** 2016-01-04

**Authors:** Muhammad Ehsaan, Wouter Kuit, Ying Zhang, Stephen T. Cartman, John T. Heap, Klaus Winzer, Nigel P. Minton

**Affiliations:** Clostridia Research Group, BBSRC/EPSRC Synthetic Biology Research Centre (SBRC), University of Nottingham, University Park, Nottingham, NG7 2RD UK; MicCell Bioservices B.V., Edisonstraat 101, 7006 RB Doetinchem, The Netherlands; Department of Life Sciences, Centre for Synthetic Biology and Innovation, Imperial College London, South Kensington Campus, London, SW7 2AZ UK; Intermediates Sustainability, INVISTA Intermediates, Wilton Centre, Redcar, TS10 4RF UK

**Keywords:** Allelic exchange, In-frame deletion, Counter selection marker, *codA*, *pyrE*, *Clostridium acetobutylicum*, Whole genome re-sequencing

## Abstract

**Background:**

*Clostridium acetobutylicum* represents a paradigm chassis for the industrial production of the biofuel biobutanol and a focus for metabolic engineering. We have previously developed procedures for the creation of in-frame, marker-less deletion mutants in the pathogen *Clostridium difficile* based on the use of *pyrE* and *codA* genes as counter selection markers. In the current study we sought to test their suitability for use in *C. acetobutylicum.*

**Results:**

Both systems readily allowed the isolation of in-frame deletions of the *C. acetobutylicum* ATCC 824 *spo0A* and the *cac824I* genes, leading to a sporulation minus phenotype and improved transformation, respectively. The *pyrE*-based system was additionally used to inactivate a putative glycogen synthase (CA_C2239, *glgA*) and the pSOL1 amylase gene (CA_P0168, *amyP*), leading to lack of production of granulose and amylase, respectively. Their isolation provided the opportunity to make use of one of the key *pyrE* system advantages, the ability to rapidly complement mutations at appropriate gene dosages in the genome. In both cases, their phenotypes were restored in terms of production of granulose (*glgA*) and amylase (*amyP*). Genome re-sequencing of the ATCC 824 COSMIC consortium laboratory strain used revealed the presence of 177 SNVs and 49 Indels, including a 4916-bp deletion in the pSOL1 megaplasmid. A total of 175 SNVs and 48 Indels were subsequently shown to be present in an 824 strain re-acquired (Nov 2011) from the ATCC and are, therefore, most likely errors in the published genome sequence, NC_003030 (chromosome) and NC_001988 (pSOL1).

**Conclusions:**

The *codA* or *pyrE* counter selection markers appear equally effective in isolating deletion mutants, but there is considerable merit in using a *pyrE* mutant as the host as, through the use of ACE (Allele-Coupled Exchange) vectors, mutants created (by whatever means) can be rapidly complemented concomitant with restoration of the *pyrE* allele. This avoids the phenotypic effects frequently observed with high copy number plasmids and dispenses with the need to add antibiotic to ensure plasmid retention. Our study also revealed a surprising number of errors in the ATCC 824 genome sequence, while at the same time emphasising the need to re-sequence commonly used laboratory strains.

**Electronic supplementary material:**

The online version of this article (doi:10.1186/s13068-015-0410-0) contains supplementary material, which is available to authorized users.

## Background

*Clostridium* species are a diverse and large grouping of anaerobic, endospore-forming bacteria of importance both to medicine and industry [1]. To many, the term ‘clostridia’ is synonymous with disease, a consequence of the exploits of such notorious human and animal pathogens as *Clostridium botulinum*, *Clostridium tetani*, *Clostridium perfringens* and *Clostridium difficile* [2]. The vast majority of the genus are, however, entirely benign and produce all manner of useful chemicals and fuels [3]. Of particular note are the saccharolytic species able to produce the biofuel *n*-butanol typified by *Clostridium beijerinckii*, *Clostridium saccharolyticum*, *Clostridium saccharoperbutylacetonicum* and *Clostridium acetobutylicum* [4]. All four species are associated with the Acetone-Butanol-Ethanol (ABE) commercial process [5], but it is the latter species that garners the most attention. Accordingly, attempts to develop tools for manipulating metabolism at the genetic level, both to understand and bring about improvements to the fermentative process, have tended to be skewed towards *Clostridium acetobutylicum* [6].

Early strategies that sought to use recombination-based methods to inactivate genes of *C. acetobutylicum* were handicapped by the lack of counter-selection markers for use in clostridia and, therefore, merely relied on the integration of the entire plasmid carrying an internal segment of the targeted gene [7–10]. As the plasmid carried a marker gene coding for resistance to an antibiotic, the desired single crossover integrant could be selected on the basis of acquisition of resistance to that antibiotic through its inclusion in the agar medium. Such mutants were intrinsically unstable, a consequence of the presence of duplicated DNA regions via which homologous recombination led to excision of the integrated mutagenic plasmid. Subsequently, researchers turned to the use of group II intron retargeting systems in which an intron-encoding segment of DNA could be deliberately inserted into the genomic target from where it could not excise [11–13]. The mutants generated were, therefore, extremely stable. Clones carrying the inserted intron-encoding DNA could be detected by appropriate PCR screening, or in the case of the ClosTron, through selection for acquisition of resistance to erythromycin due to the incorporation of an inactivated *ermB* gene, or RAM (Retrotransposition Activated Marker), that became functional as a consequence of the successful insertion of the group II intron-encoding DNA into the genomic target [14–16].

The efficiency, reproducibility, speed and ease of use have led ClosTron technology to become the most widely used clostridial mutagen [17]. As with any insertional tool, however, its use is disadvantaged by the possible occurrence of polar effects on downstream genes which can complicate the subsequent analysis of mutant phenotypes. As a consequence, marker-less, in-frame deletions generated by homologous recombination are preferred. Accordingly, in the past few years a number of groups have developed such methods through the successful deployment of a range of counter-selection markers first exemplified in other microbial species. A number of the markers first reported could not be used in a standard wild-type host, but required a specific mutant background. These included the genes *galK* (galactokinase), *pyrF* (5-phosphate decarboxylase) and *upp* (uracil phosphor-ribosyltransferase), exemplified in *C. perfringens* [18], in *Clostridium thermocellum* [19] and in *C. acetobutylicum* [20], respectively. Thereafter, a number of alternative counter selection markers have been described that can be used in a wild-type background, most notably use of the *E. coli* genes *codA* (cytosine deaminase) and *mazF* (mRNA interferase) in *C. difficile* [21] and *C. acetobutylicum* [22], respectively, and the exploitation of the *Thermoanaerobacterium saccharolyticum tdk* (thymidylate synthetase) and *C. thermocellum hpt* (hypoxanthine phosphoribosyl transferase) genes to make knock-outs in *C. thermocellum* [23].

Of particular significance was the development of a method, now termed Allele-Coupled Exchange (ACE), which allows the rapid insertion of heterologous DNA, without inherent limits on size or complexity, into the genome [24]. Following integration of the plasmid by single-crossover recombination, the system is designed such that during the desired second recombination event, a plasmid borne allele becomes ‘coupled’ to a genome located allele which leads to the creation of a new selectable allele, allowing the isolation of double-crossover cells. The use of highly asymmetric homology arms dictates the order of recombination events. A long homology arm directs the first recombination event (plasmid integration) and a much shorter homology arm directs the second recombination event (plasmid excision). Whilst a number of different genetic loci may be used to insert heterologous DNA via ACE, one exemplification of the method exploits the native *pyrE* gene, bringing about its inactivation by replacement of the wild-type allele with a mutant allele lacking approximately 300 bp from the 3′ end of the structural gene. The *pyrE* gene encodes orotate phosphoribosyltransferase [E.C.2.4.2.10] which, in common with PyrF, is an enzyme involved in *de novo* pyrimidine biosynthesis. It may be used as a positive/negative selection marker as it is essential in the absence of exogenous pyrimidines and renders 5-fluoroorotate (FOA) toxic to cells. It follows that a heterologous *pyrE* gene can be used as a counter-selection marker in such a strain, in an equivalent manner to *pyrF* [19], a facility that was demonstrated in two different strains of *C. difficile* using a heterologous *pyrE* allele from *Clostridium sporogenes* [25]. Crucially, however, the design of the created *pyrE* mutant strain is such that its *pyrE* allele can be rapidly (2 days) restored to wild-type using an appropriate ACE correction vector allowing the specific in-frame deletion mutant to be characterised in a clean, wild-type background. Moreover, this facility provides the parallel opportunity to complement the mutant at an appropriate gene dosage, through insertion of a wild-type copy of the gene, either under the control of its native promoter or the strong P_fdx_ promoter (derived from the ferredoxin gene of *Clostridium sporogenes*), concomitant with restoration of the *pyrE* allele back to wild-type [25].

In the present study we have extended the significant advantages of the *pyrE*-based system to *C. acetobutylicum* through appropriate deployment of ACE. At the same time, we took the opportunity to test the suitability of using *codA* as a counter selection marker. During the course of this study, genome sequencing of the strains used revealed the presence of a significant number of SNVs (Single Nucleotide Variations) and Indels (Insertions and Deletions) compared to that expected, emphasising the need for researchers to both establish the sequence of the strains in use in their laboratory and to appropriately curate their laboratory strains.

## Results

### Establishment of *pyrE* and *codA* Knock-out Vectors for use in *C. acetobutylicum*

**Fig. 1:**
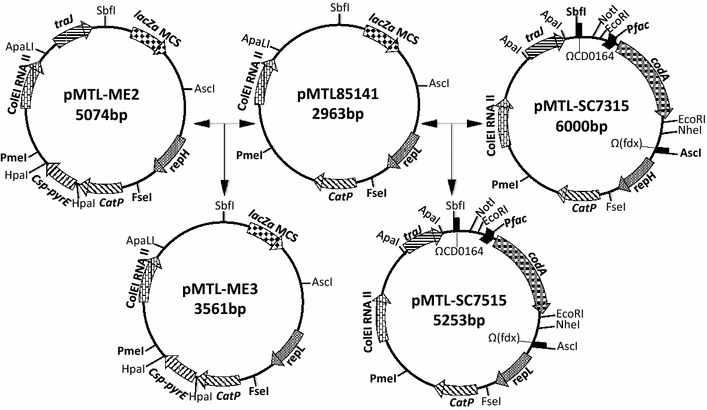
Clostridial KO vector pMTL-ME3 and pMTL-SC7515. Plasmid components are: CatP, the *catP* gene of *C. perfringens* conferring thiamphenicol resistance; Csp-*pyrE*, the *pyrE* gene of *C. sporogenes*; ColE1 RNAII, the replication region of the *E.coli* plasmid ColE1; *traJ*, transfer function of plasmid RP4 *oriT* region; ΩCD0164, a transcriptional terminator isolated from downstream of the *C. difficile* strain 630 CD0164 gene; lacZα MCS, the *lacZ’* gene encoding the alpha fragment of the *E.coli* β-galactosidase, containing a multiple cloning site, MCS, region derived from plasmid pMTL20; Ω(fdx), a transcriptional terminator of the ferredoxin gene of *C. pasteurianum*; *repL* is the replication protein of plasmid pIM13; *codA*, the cytosine deaminase gene of *E. coli*, and; the promoter in front of *codA* is the P_fac_ promoter of pMTL007

The use of *pyrE* as a counter selection marker in *C. acetobutylicum* required the construction of a knock-out (KO) vector suited to this clostridial species and the availability of an appropriate *pyrE* mutant host. The required *pyrE* (CA_C0027) mutant (CRG1545), lacking approximately 300 bp from the 3′-end of the gene, has been previously made using ACE [24]. The existing *pyrE*-based, KO plasmid, pMTL-YN3 [25], used for making allelic exchange mutants in *C. difficile* 630 is based on a plasmid replicon (that of pCB102 [26]) shown to be replication defective in this particular clostridial strain [25]. Plasmid pMTL-YN3 additionally carries a functional copy of the *pyrE* gene of *C. sporogenes* ATCC 15579 that had been transcriptionally fused to a *C. perfringenscatP* gene [27]. As the pIM13 replicon has previously proven most useful for allelic exchange vectors in *C. acetobutylicum* [24], the pCB102-derived [26] replicon of plasmid pMTL-YN3 was replaced with that of pIM13 (Methods) and the plasmid obtained designated pMTL-ME3 (Fig. 1). To test that the *C. sporogenes* ATCC 15579 was functional in *C. acetobutylicum,* CRG1545 was transformed either with plasmid pMTL-ME3 or a vector control lacking *pyrE*, pMTL85141. Transformants were selected on CGM supplemented with 15 μg/ml thiamphenicol (Tm) and 20 μg/ml uracil and incubated for 24 h. Single colonies were re-streaked onto the same medium followed by streaking onto CBM agar, without uracil. All the cells transformed with pMTL-ME3 showed growth similar to that of the *C. acetobutylicum* ATCC 824 wild-type but the cells transformed with the control vector pMTL85141 and the plasmid-free CRG1545 grew very poorly on minimal medium (Additional file 4: Fig. S1). These data confirmed that the *C. sporogenes* ATCC 1557 *pyrE* gene was functional in *C. acetobutylicum* ATCC 824 and could most likely be used as a counter selection marker.

Similar to pMTL-YN3, the previously constructed *codA*-based KO vector pMTL-SC7315 used in *C. difficile* [21] is based on the replicon of pCB102 [26]. Accordingly, its replication region was also changed to that of pIM13 (Methods) to give the plasmid pMTL-SC7515 (Fig. 1). Cytosine deaminase (EC 3.5.4.1) converts the innocuous pyrimidine analogue 5-fluorocytosine (FC) into the highly toxic 5-fluorouracil (FU). FU toxicity occurs via uracil phosphoribosyltransferase (EC 2.4.2.9), followed by a series of steps that result in irreversible inhibition of thymidylate synthase, a key enzyme in nucleotide biosynthesis, and misincorporation of fluorinated nucleotides into DNA and RNA [28, 29]. As the genome of *C. acetobutylicum* ATCC 824 [30] lacks any obvious *codA* gene but does carry a homologue of *upp* (CA_C2879), it would be predicted that the introduction of a functional *codA* gene should result in heightened sensitivity to FC. To test this assumption, the ATCC 824 wild-type was independently transformed with the *codA-*based plasmid pMTL-SC7515 and the vector pMTL85151 [31] and the sensitivity of the resultant transformants to FC assessed. The MIC of FC on CBM was reduced from 1 mg/ml (for the wild-type and pMTL85151 empty-vector control strain) to 100 µg/ml in those cells carrying pMTL-SC7515 (data not shown). This confirmed that *codA* could most likely form the basis of a counter selection marker in *C. acetobutylicum*.

### Establishment of ACE *pyrE* correction, complementation and overexpression vectors

A set of three ACE vectors were previously [24] established for use in *C. difficile* knock-out mutants that provide the facility to either (1) simply ‘correct’ the *pyrE* allele of the host back to wild-type (a so-called *pyrE ‘*correction’ vector), thereby allowing the establishment of a KO phenotype of a gene of interest in an otherwise wild-type background; (2) provide cloning sites downstream of the *pyrE* allele that allow a functional copy of the KO gene, together with its own promoter, to be inserted into the genome concomitant with correction of the *pyrE* allele, thereby allowing complementation at an appropriate gene dosage and (3) provide cloning sites that are preceded by the strong P_*fdx*_ promoter that facilitate overexpression of the complementing gene. In *C. difficile* 630, these vectors were designated pMTL-YN1, pMTL-YN1C and pMTL-YN1X, respectively [23]. Accordingly, equivalent vectors were made for *C. acetobutylicum*, namely pMTL-ME6, pMTL-ME6C and pMTL-ME6X (Methods). As a quick check to establish that each vector was capable of correcting the *pyrE* allele, all three were independently transformed into CRG1545 and plated on CGM medium containing 20 µg/ml uracil and 15 µg/ml Tm, and selected colonies restreaked onto CBM lacking uracil and any antibiotic supplementation. In all cases, prototrophic colonies were isolated consistent with the correction of the *pyrE* allele.

### Creation of mutants using *codA* as negative selection marker

To demonstrate the utility of *codA* as a counter selection marker in the generation of KO mutants, two genes were targeted, namely *spo0A* and the *cac824I* encoding the principal type II restriction system of *C. acetobutylicum.* Appropriate KO cassettes, essentially comprising approximately 750 bp from upstream and downstream of the target gene, were assembled and cloned into pMTL-SC7515 as described in “Methods”. The two plasmids generated (pMTL-SC7515::*spo0A* and pMTL-SC7515::*cac824I*) were transformed into the wild-type strain and the procedure described in “Methods” implemented. This involved initially purifying single crossover integrants (through the selection of large, faster-growing transformants), transferring to un-supplemented CGM medium for 2–3 days to allow cells to undergo a second recombination event and then selecting FC-resistant cells on CBM agar plates supplemented with 100 µg/ml FC. All of those cells that retain the *codA* gene (either those still containing the autonomously replicating plasmid, or those in which the plasmid had integrated) are sensitive to FC and are, therefore, unable to grow. In contrast, those cells in which the plasmid has excised, through a second recombination event, and has been lost due to segregational instability, are able to grow in the presence of FC. The loss of the KO vector was confirmed by replica plating on CGM with or without Tm supplementation.

**Fig. 2:**
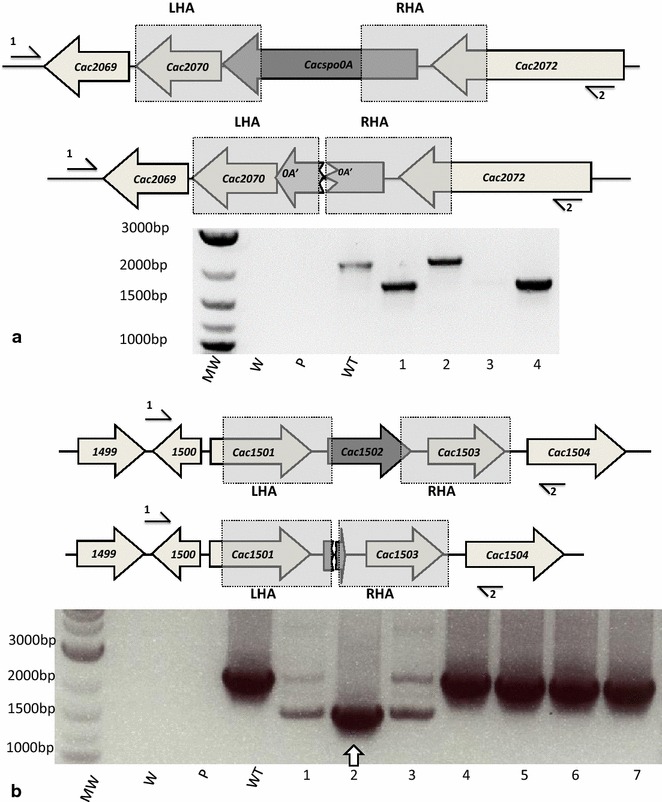
Isolation of in-frame deletion mutants of *spo0A* and *cac824I.* Schematic representations of the wild-type and mutant genomes in the targeted regions are shown above each electrophoretogram, together with the relative position of the two primers used and the extent of the Left Homology Arm (LHA) and Right Homology Arm (RHA) employed to mediate recombination. In both: MW, 2-log DNA marker (NEB) molecular weight marker; ‘W’, is a PCR with no DNA; ‘P’, is a PCR using the plasmid only, and; ‘WT’ is the wild-type strain CRG1268. **a** PCR screening of four FC^R^ colonies (labelled *lanes* *1*–*4*) using primers Cac-spo0A-sF2 (*1*) and Cac-spo0A-sR2 (*2*). Expected fragment sizes are 1679 bp in the deletion mutant and a 2114 bp fragment in the wild-type, respectively. Accordingly, labelled* lane* WT is wild-type, *lanes 1, 3* (very weak band) and *4* are mutants. **b** PCR screening of seven FC^R^ colonies (labelled* lanes* 1–7) using primers Cac-1501-sF2 (*1*) and Cac-1504-sR1 (*2*). Expected fragment sizes are 1611 bp in the deletion mutant and a 2319 bp fragment in the wild-type, respectively. Accordingly, labelled *lanes 4–7* are wild-type, *lane 2* is a mutant, and *lanes 1* and *3* are a mixture of mutant and wild-type

In the case of the experiment that targeted *spo0A,* of the four random, FC^R^ colonies screened by PCR using primers flanking the gene (Cac-spo0A-sF2 and Cac-spo0A-sR2), one generated a DNA fragment of a size (2114 bp) equivalent to the wild-type, the other three generated a 1679-bp DNA fragment consistent with the presence of the desired deletion (Fig. 2a). Nucleotide sequencing of the later fragments confirmed the presence of the expected in-frame deletion. Consistent with a sporulation defect, all three putative mutants were shown by microscopy to lack phase-bright spores and were unable to form colonies after being subjected to heat treatment at 80 °C for 10 min and plating on CGM agar and incubation for 24 h (data not shown).

In the experiment designed to KO *cac824I*, of the seven FC^R^ colonies screened only one of them (clone 2) was confirmed as an in-frame deletion mutant, by generation of an appropriately sized fragment with the correct sequence (Fig. 2b). Four of the clones (clones 4–7) appeared to have reverted back to wild-type whereas 1 and 3 appeared to be composed of a mixture of both wild-type and the in-frame deletion mutant. Insertional inactivation or deletion of *cac824I* by other groups has led to the observation that mutant cells can be transformed with unmethylated DNA as efficiently as with methylated plasmid DNA [32, 33]. Consistent with this finding, 1.5 × 10^4^ transformants per µg DNA were obtained when *Δcac824I* was transformed with unmethylated plasmid compared to wild-type where no colonies were obtained.

### In-frame deletion of *spo0A* and its complementation using *pyrE* correction vectors

**Fig. 3:**
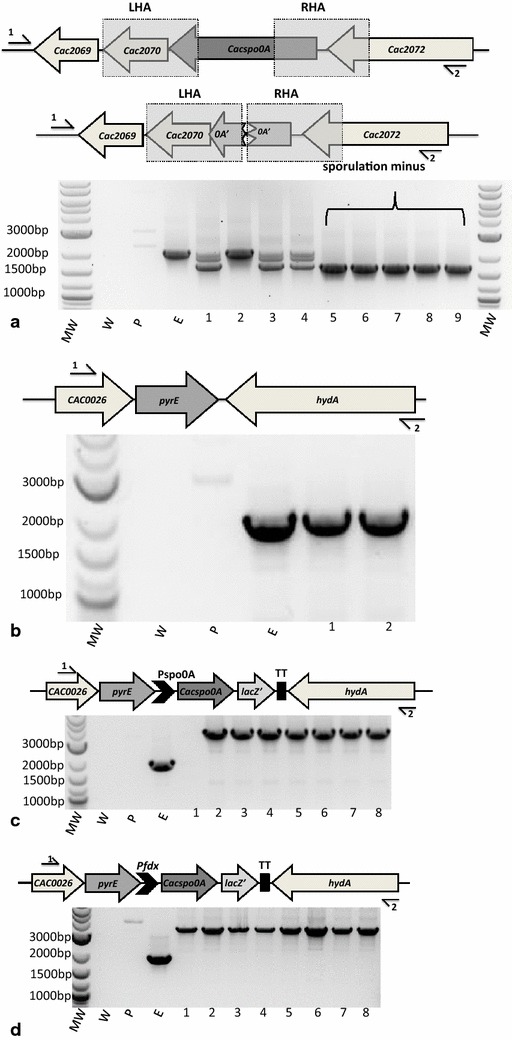
Screening of Spo0A mutants and ACE corrected/complemented/overexpressed clones. *Key* MW, 2-log DNA marker (NEB) molecular weight marker; ‘W’, is a PCR with no DNA; ‘P’, is a PCR using the plasmid only, and; ‘E’ is the *pyrE* minus parent strain CRG1545. On **b**, **c** the final arrangement of the chromosomal region in the *pyrE* repaired strains is illustrated. Components are: *dcd,* deoxycytidine triphosphate deaminase; *cac0026*, hypothetical protein; *pyrE,* orotate phosphoribosyltranspherase; P_spo0A_, promoter of the *spo0A* gene; P_fdx_, promoter of the *C. pasteurianum* ferredoxin gene; *spo0A*, encodes the master regulator of sporulation, Spo0A; *lacZ’,* alpha-peptide of β-galactosidase gene containing multiple cloning sites; TT, transcriptional terminator found between *hydA* (hydrogenase) and *pyrE*. *Arrows* above and below the sequence labelled *1* and *2* show position of primers specific to each experiment. *Lanes* *1*–*9* (**a**), *1*–*2* (**b**) and *1*–*8* (**c**, **d**) are the screened DNA samples from randomly selected uracil prototrophic clones. **a** PCR screening of nine FOA^R^ colonies using flanking primers Cac-spo0A-sF2 (*1*) and Cac-spo0A-sR2 (*2*). The expected PCR product for the mutant is 1679 bp, for the parent strain CRG1545 is 2114 bp. **b** PCR screening of the two uracil prototroph clones using flanking primers Cac0026-sF2 (*1*) and Cac-hydA-sR2 (*2*). The expected PCR product for the *pyrE* mutant parent strain is 1936 bp and for the strain in which the *pyrE* allele has been restored to wild-type with pMTL-ME6 is 1989 bp. **c** PCR screening of eight uracil prototroph using flanking primers Cac0026-sF2 (*1*) and Cac-hydA-sR2 (*2*). The expected PCR product for the parent *pyrE* minus strain is 1936 bp and for the strain in which the *pyrE* allele has been restored to wild-type with pMTL-ME6C::*spo0A* is 3610 bp. **d** PCR screening of eight uracil prototroph using flanking primers Cac0026-sF2 (*1*) and Cac-hydA-sR2 (*2*). The expected PCR product for the *pyrE* mutant parent strain is 1936 bp and for the strain in which the *pyrE* allele has been restored to wild-type with pMTL-ME6X::*spo0A* is 3450 bp

In parallel to the experiments with the *codA*-based vector pMTL-SC7515, the equivalent *spo0A* KO cassette was cloned into the *pyrE*-based KO vector pMTL-ME3, transformed into the *pyrE* mutant *C. acetobutylicum* strain CRG1545 and subjected to the mutant isolation procedure outlined in “Methods”. After two passages of nine independent Tm^R^ transformants on CGM supplemented with 15 µg/ml Tm, three of the visible larger colonies were single crossover integrants (two RH, one LH). Single crossover integrants were streaked onto CGM medium supplemented with 20 µg/ml uracil and incubated for 2–3 days to allow cells to undergo a second recombination event. Bacterial cell suspensions in PBS were spread on CGM supplemented with 400 µg/ml FOA and 1 µg/ml uracil. The loss of the vector in those cells that grew was confirmed by demonstration that they could no longer grow when patch plated onto CGM medium containing 15 µg/ml Tm. A total of nine randomly selected Tm^S^ clones were screened using *spo0A* flanking PCR primers Cac-spo0A-sF2 and Cac-spo0A-sR2. A DNA fragment of a size (2114 bp) equivalent to the wild-type was obtained with one clone; five clones generated a 1679-bp fragment consistent with the presence of the desired deletion while the remaining three generated both a wild-type and mutant DNA fragment (Fig. 3a). Nucleotide sequencing of the 1679-bp fragment of the five mutants confirmed the presence of the in-frame deletion.

**Fig. 4:**
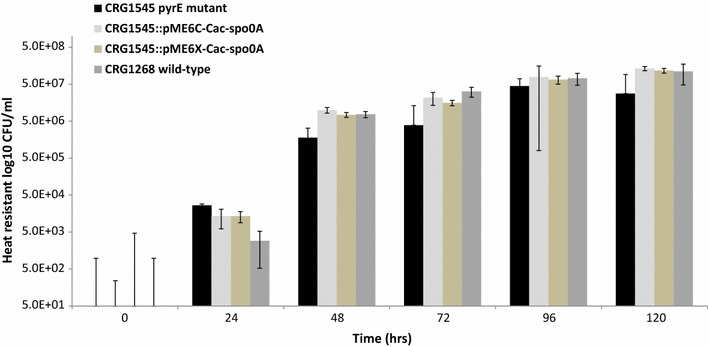
Sporulation frequency of the *spo0A* mutant and ACE-complemented derivatives. The various strains were cultivated in CBMS medium at 37 °C and samples taken at 0, 24, 48, 72, 96 and 120 h, subjected to a heat shock (80 °C for 10 min) before plating at serial dilutions on CBM agar and incubated for 48 h. The number of colonies was counted and heat resistant Colony Forming Units per ml (CFU/ml) was calculated. Estimates were undertaken in triplicate. The detection limit of the assay is 50 CFU/ml, so no counts were recorded with the *spo0A* mutant control (not shown) or at 0 h with any of the strains

Prior to measurements of their phenotype, the *pyrE* allele of all the mutants was corrected back to wild-type using the ACE correction vector pMTL-ME6 (Fig. 3b). Their sporulation minus phenotype was confirmed by the demonstration that none formed phase-bright spores after 2 to 5 days growth in CBM supplemented with CaCO_3_ and that they could no longer form CFU when plated on CGM after an 80 °C heat shock for 10 min (Fig. 4). In parallel to correction of the *pyrE* locus, complementation and overexpression experiments were undertaken with one of the mutants, CRG3520. Accordingly, the *spo0A* gene together with its own promoter was cloned into pMTL-ME6C (yielding the pMTL-ME6C::*spo0A*) and into plasmid pMTL-ME6X downstream of the strong P_*fdx*_ promoter [14], to give plasmid pMTL-ME6X::*spo0A*. The two plasmids were transformed into CRG3520, selecting for Tm^R^, and then restreaked onto CBM media lacking uracil to select for clones in which the *pyrE* gene had been restored to wild-type. Confirmation that in both instances the *spo0A* gene had become integrated downstream of repaired *pyrE* gene was obtained using the flanking primers Cac0026-sF2 and Cac-hydA-sR2 (Fig. 3c, d). Those mutant cells in which *spo0A* was inserted downstream of the corrected *pyrE* locus under the transcriptional control of its native promoter or the P_*fdx*_ promoter were completely restored to a wild-type phenotype, in terms of the presence of phase-bright spores in mature cultures and CFU after heat shock. An estimate of the number of spores produced by the various cultures is shown in Fig. 4.

Although overexpression of *spo0A* using P_*fdx*_ promoter did not show any change in the sporulation frequency, a clear phenotypic change was observed as colonies of the overexpressed strain were visibly bigger compared to complemented and wild-type strains when grown on CBM medium after 24 h (Additional file 4: Fig. S2).

### In-frame deletion of the α-amylase gene and its complementation using *pyrE*

To further test the system, and exemplify the utility of overexpressing complementing genes at the *pyrE* locus in mutant strains, it was desirable to use a mutant which had an easily visualised phenotype. An ideal candidate would be a secreted enzyme for which there is a simple plate-based assay that could measure zones of hydrolysis around a colony. Production of pectinase was initially considered to be an ideal candidate. To rapidly identify such a gene, intron insertion mutants were made using ClosTron technology [14] in CA_C0355, annotated as encoding polygalacturonase/pectinase, at two distinct insertion sites, 656a and 1167 s. No difference was observed in the size of the halo formation in the mutants compared to wild-type after staining of plates with iodine [34] (data not shown). This indicated either the targeted gene was not responsible for the observed pectinase activity in the assay used or, more likely, more than one enzyme has such an activity.

As an alternative, the pSOL1 *amyP* gene (CA_P0168) was selected which is responsible for the production of an extracellular α-amylase when grown on medium containing glucose as the sole carbon source. The expression of this gene could be easily assessed using CBM plates containing starch and stained with iodine. Loss of the pSOL1 megaplasmid, and by consequence *amyP*, is known to result in a loss of halo formation on medium containing starch [34]. This phenotype was confirmed by the initial generation of a ClosTron mutant, which showed a dramatic reduction in visible halo on starch iodine plates (data not shown). Accordingly, the *C. acetobutylicum* 824 *pyrE* mutant was transformed with vector pMTL-ME3::*amyP* (Methods), and the in-frame deletion procedure followed exactly as described for *spo0A*. Three FOA^R^ colonies were screened for the putative mutation which exhibited a reduction in halo size on starch iodine plates of an equivalent size to that seen with the ClosTron mutant. PCR screening of these clones using primers flanking the amylase gene (Cac-amyP-sF2 and Cac-amyP-sR2) resulted in the expected sized (2078 bp) DNA fragment (Additional file 4: Fig. S3A). Nucleotide sequencing of this 2078-bp fragment confirmed the presence of the intended in-frame deletion.

**Fig. 5:**
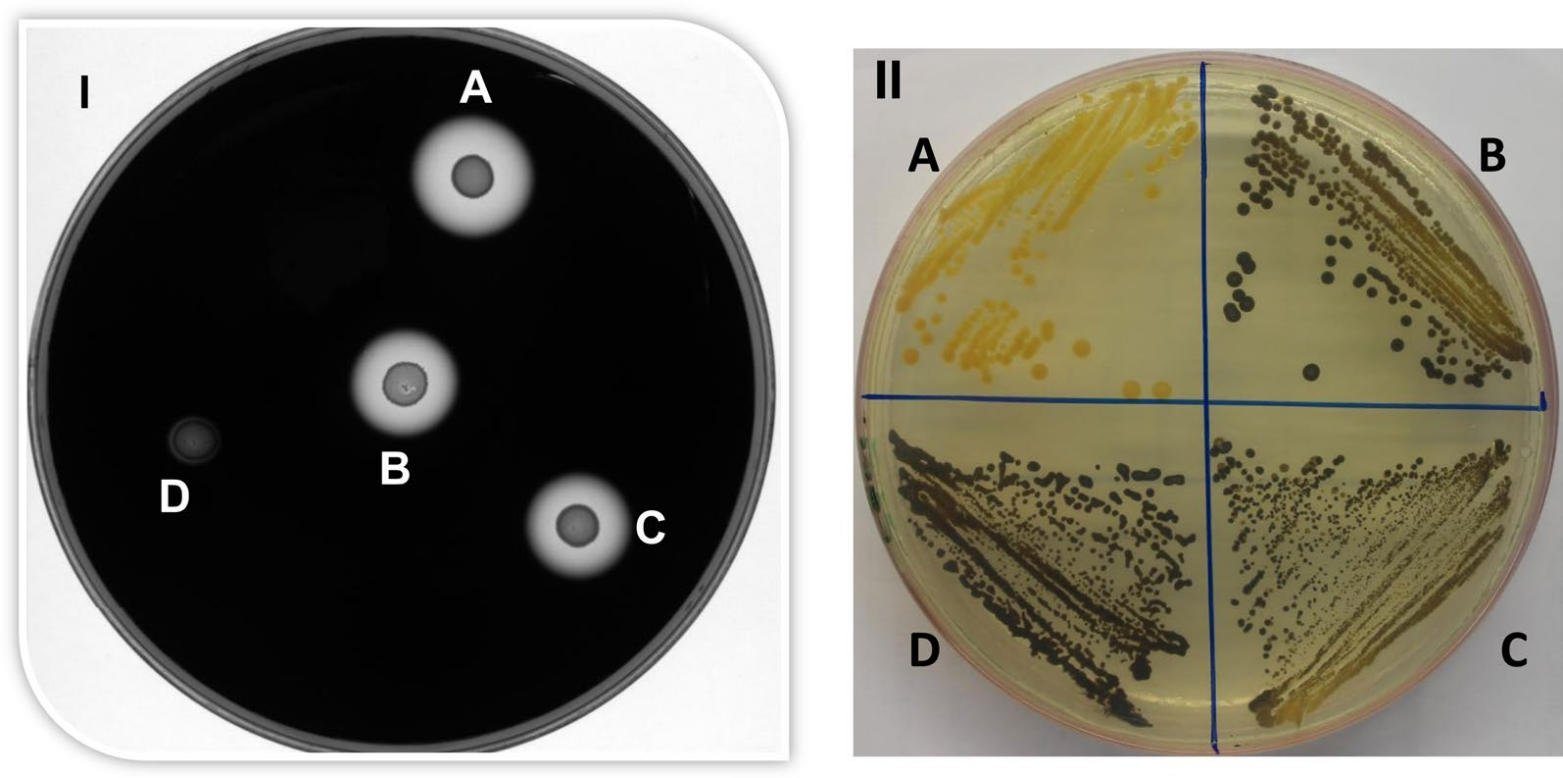
Phenotypes of the amylase and granulose mutants and complemented clones. **I** A 20 µl aliquot of a normalised overnight culture was spotted on CBM agar containing 2 % (w/v) starch, 0.5 % Glucose and plate stained with iodine potassium iodide solution after 48 h. *A* CRG4897, carrying a functional copy of *amyP* under the control of the P_fdx_ promoter inserted into the genome using the ACE vector pMTL-ME6X::*amyP*. *B* *C. acetobutylicum* ATCC 824 COSMIC strain wild-type. *C* CRG4896, carrying a functional copy of *amyP* and its own promoter inserted into the genome using the ACE vector pMTL-ME6C::*amyP*. *D* CRG4894, Δ*amyP* mutant. **II** All strains were grown on CBM agar plates with 5 % glucose to promote granulose formation and exposed to iodine vapours after 72 h. *A* CRG4898, Δ*glgA* mutant; *B* CRG4901 carrying a functional copy of *glgA* and its own promoter inserted into the genome using the ACE vector pMTL-ME6C::*glgA*; *C* CRG4902 carrying a functional copy of *glgA* under the control of the P_fdx_ promoter inserted into the genome using the ACE vector pMTL-ME6X::*glgA*. *D* *C. acetobutylicum* ATCC 824 COSMIC strain wild-type

A single mutant was selected and its *pyrE* allele was repaired, complemented and overexpressed using the ACE vectors pMTL-ME6, pMTL-ME6C::*amyP* and pMTL-ME6X::*amyP*, respectively, as described for the *spo0A* mutant. All the strains generated, together with the mutant and wild-type control, were grown overnight in 2x YTG broth. ODs were normalised and 20 µl of each culture was spotted on CBM agar plates containing 2 % starch and 0.5 % glucose and incubated for 48 h in an anaerobic cabinet. After 48 h, the plate was stained with iodine potassium iodide solution (Sigma 32922) for 1 min and photographed. The results (Fig. 5I) showed that there is no halo formation in the case of the mutant and size of halos is similar in the case of wild-type and complemented strain while slightly bigger halo was evident in the case of the overexpression strain.

### In-frame deletion of the *glgA* gene and its complementation using *pyrE*

As a final test of the utility of the *pyrE* KO system, a mutation predicted to cause a defect in granulose accumulation was targeted. Granulose is a starch-like storage compound that is accumulated in late exponential/stationary phase prior to sporulation and acts as a carbon and energy source for subsequent endospore formation. It is formed via the ADP-glucose pathway which requires ADP-glucose pyrophosphorylase (EC 2.7.7.27) and granulose synthase (EC 2.4.1.21) [35, 36]. The *C. acetobutylicum* ATCC 824 genome contains a cluster of genes involved in the glycogen formation (CA_C 2237- CA_C 2239), that includes CA_C2239 (*glgA*) predicted to encode a glycogen synthase responsible for the formation of alpha-1,4-glucan chains from ADP-glucose [29]. Accordingly, a KO cassette directed against *glgA* was assembled and cloned into pMTL-ME3, and the resultant plasmid pMTL-ME3::*glgA* used to isolate an in-frame deletion mutant in CRG1545 using the standard protocol (Methods). In this instance, PCR screening of six random FOA^R^ clones using the flanking primers Cac-glg-sF2 and Cac-glg-sR1 generated a DNA fragment from two of a size equivalent to that obtained with the wild-type (3112 bp), while two clones generated a DNA fragment of a size (1684 bp) consistent with that which would be obtained with a deletion mutant (Additional file 4: Fig. S4). PCR failed for the remaining two clones. Nucleotide sequencing of the 1684 bp fragment confirmed the presence of the intended in-frame deletion.

A single mutant was selected and its *pyrE* allele repaired using pMTL-ME6, pMTL-ME6C::*glgA* and pMTL-ME6X::*glgA*, the latter two plasmids corresponding to pMTL-ME6C carrying the *glgA* gene together with its native promoter and pMTL-ME6X carrying *glgA* under the control of the P_*fdx*_ promoter, respectively. The three clones generated were then tested for granulose accumulation using CBM agar plates containing 5 % glucose and exposure to for 1 min to iodine vapour following growth for 72 h (Fig. 5II). These data confirmed that the *glgA* mutant no longer produced granulose and that its production was confirmed in both complemented strains. Any effect of placing the gene downstream of the strong P_*fdx*_ promoter was not discernible using this test.

### High-throughput sequencing of the *pyrE* and parent strains

Having generated the *pyrE* mutant CRG1545 to be used as the host for future mutational studies with pMTL-ME3, it was important to establish that no major changes had occurred within the genome. The genome of the *pyrE* mutant strain (CRG1545) together with its parent strain (CRG1268) were, therefore, subjected to Illumina, single-read sequencing and the data obtained mapped to the reference GenBank sequence NC_003030 (chromosome) and NC_001988 (pSOL1) using CLC Genomics Workbench. Surprisingly, a total of 177 SNVs and 49 Indels were shared by the parent and its *pyrE* mutant (Additional file 2: Table S2). Aside from the presence of the expected *pyrE* deletion, the *pyrE* deletion strain appeared to contain one additional SNV not present in the parent strain (Table 2). This equated to a G > A substitution in CA_C3553 at position 3749347, which caused an Arg > Lys change at amino acid position 83 of an encoded LacI family transcriptional regulator. The presence of this change in the *pyrE* mutant (CRG1545), and not the progenitor CRG1268, was confirmed by the amplification of a region of DNA encompassing the SNV and subjecting it to Sanger sequencing.

For comparative purposes, ATCC 824 was re-acquired from the ATCC (November 2011) and designated as CRG3286, genome DNA prepared and subjected to Illumina single-read re-sequencing. Of the 177 SNVs and 49 Indels common to the ATCC 824 COSMIC consortium strain (CRG1268) and its *pyrE* mutant (CRG1545), 175 and 48 (Additional file 2: Table S2), respectively, were also present in the newly acquired ATCC 824 genome (CRG3286). Of the two SNVs unique to CRG1268 and CRG1545, one occurred within CA_C1534 encoding an Iron ABC transporter ATP-binding protein and resulted in an amino acid substitution at position 303. The other resided just upstream of CA_C3087 (phosphoenolpyruvate-protein kinase). The remaining deletion comprised a 4916-nt region of the pSOL1 megaplasmid (position 69715 to 74631, NC_001988) (Table 3). This resulted in the deletion of four genes, namely *manY*/*levF* (mannose/fructose-specific PTS comp. IIC), *ptnd* (mannose-specific PTS comp. IID), CA_P0069 (hypothetical protein) and CA_P0070 (HAD phosphatase superfamily protein) and partial deletion of *ptna* (mannose-specific PTS component IIAB) and CA_P0071 (xylan degrading enzyme). The region deleted is flanked by a 10-bp sequence, TGACAACCAG at positions 69705–69714 and 74622–74631, a repeat sequence that most likely mediated the 4916-bp deletion. A closer examination of the mapping data for the ATCC 824 (COSMIC) Illumina reads against the pSOL1 sequence (NC_001988) revealed that the deleted region was present, but at a very low coverage (an average read occurrence of approximately 20 compared to 400 for the remainder of pSOL1).

As the presence of the large pSOL1 deletion in the *pyrE* strain was not ideal, we opted to remake the *pyrE* mutant in the re-acquired ATCC 824 strain. Accordingly, the ACE vector pMTL-JH12 was used to remake the mutant as originally described [24]. The new mutant strain was designated CRG3899 and a paired-end library constructed from its genome and re-sequenced using Illumina technology. The new strain carried the same 175 SNVs and 48 Indels common to the ATCC 824 re-acquired parent strain, the COSMIC consortium strain and its *pyrE* mutant, but did not contain the ca. 5 kb deletion present in pSOL1 or the CA_C3553 SNV found in the previous *pyrE* mutant (CRG1545). The re-sequencing analysis of the genome indicated that there were no additional SNVs or Indels compared to the parental wild-type strain.

## Discussion

**Fig. 6:**
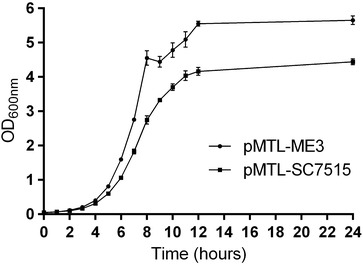
Growth curve comparison of pMTL-ME3 and pMTL-SC7515. Growth of *C. acetobutylicum* ATCC 824 in CBMS medium supplemented with 7.5 µg/ml of Tm transformed with vectors pMTL-ME3 and pMTL-SC7515 with *error bars* representing the standard error of the mean (SEM) (n = 3)

In the current study we have sought to extend two methods developed for making in-frame deletions in the important human pathogen *C. difficile* to the industrially important solvent producer, *C. acetobutylicum.* Both rely on the principle of pseudo-suicide [25], in which plasmids encoding an antibiotic resistance gene (*catP*, specifying resistance to Tm) are employed that are replication defective and limit the growth rate of the population that harbours them in the presence of antibiotic (Tm). In contrast, cells in which the plasmid has integrated by single crossover recombination grow faster and produce larger colonies, because all of the progeny carry a copy of *catP*. Following the isolation of the pure single cross-over integrants, cells are streaked onto media containing the counter selection agent (FOA or FC) to enable isolation of the double crossover mutant. The *codA*-based pMTLSC7515 uses FC, and the *pyrE*-based pMTL-ME3 uses FOA. As the two procedures are essentially the same, they would be expected to be equally effective in their ability to isolate mutants. Nonetheless, it generally proved easier to isolated ‘pure’ single-crossover, integrant colonies using pMTL-SC7515 compared to pMTL-ME3, because the larger, faster growing colonies were more readily apparent. Although both vectors employed are based on the unstable replicon of plasmid pIM13 [31], the growth rate of *C. acetobutylicum* carrying pMTLSC7515 in the presence of antibiotic is slightly lower than that of *C. acetobutylicum* carrying pMTL-ME3, and the culture achieves a lower final OD (Fig. 6). This is most likely a consequence of the larger size of pMTLSC7515 (5253 bp) compared to pMTL-ME3 (3561 bp), leading to a reduced replication efficiency.

For the isolation of double crossover mutants, the pure single crossover clones were plated onto media containing either FOA (pMTL-ME3) or FC (pMTL-SC7515). As the two plasmids are based on the same replicon and incorporate the same KO cassette for each targeted gene, they should be equally effective and the same number of mutants obtained. However, less screening was required to isolate mutants using pMTL-ME3 and FOA as fewer total colonies were generated suggesting that the rate of spontaneous mutants resistant to FOA was less than those that arose that were resistant to FC. Indeed, the majority of the FC colonies that arose still retained Tm resistance, even after several subcultures onto agar medium, suggesting that they retained an integrated plasmid and that spontaneous mutations elsewhere (i.e., in *codA, upp*) had led to acquisition of resistance to FC.

The primary aim of this study was the exemplification of the methods in an industrially relevant clostridia, so a number of the genes chosen for deletion had already been inactivated in the past. Thus, both systems readily allowed the isolation of in-frame deletions of *spo0A* (master regulator of sporulation), previously mutated using ClosTron technology and shown to abolish sporulation [13]. Similarly, an in-frame deletion of the gene CA_C1502 encoding the type II restriction endonuclease *cac824I* was created using pMTL-SC7515 and shown, as previously [32], to allow *C. acetobutylicum* to be transformed with unmethylated plasmid DNA. The *pyrE*-based system was additionally used to inactivate for the first time a putative glycogen synthase (CA_C2239, *glgA*) and the pSOL1 amylase gene (CA_P0168, *amyP*). In both cases the expected phenotype was obtained, in terms of lack of production of granulose and amylase, respectively. As these genes had not been previously inactivated, they provided the opportunity to make use of one of the key advantages of the *pyrE* system, the ability to rapidly complement mutations at an appropriate gene dosage in the genome. In both cases, their *glgA* and *amyP* phenotypes were restored in terms of production of granulose and amylase.

In parallel, the opportunity was undertaken to assess the effect of overexpressing each complementing gene using the strong P_*fdx*_ promoter. There was a discernible increase in the halo around the colony in which the genome integrated copy of *amyP* was under the transcriptional control of P_*fdx*_ (Fig. 5I, A), in comparison to either the wild-type (Fig. 5I, B) or *amyP* combined with its native promoter (Fig. 5II, C). In contrast, no discernible difference could be seen in the simple plate assay used when the *glgA* gene was overexpressed. This is perhaps not surprising, as unlike amylase degradation of starch, there is more than one step in granulose formation, and granulose synthase may not represent the rate limiting step. During complementation of *spo0A*, the effect of overexpressing *spo0A* using P_*fdx*_ was also explored. No increase/decrease in the number of spores made compared to when expression was from the native promoter was observed, although intriguingly, the colonies of the former were visibly larger (Fig. 5II). The reason for this change in colony morphology remains unknown.

The utility of *pyrE* clostridial mutants has been further demonstrated in the present study. Crucially, the mutants made are always characterised in an otherwise wild-type background as the *pyrE* mutant is always converted back to prototrophy, using ACE, prior to phenotypic characterisation. This is in contrast, for instance, to the use of a *pyrF* mutant and corresponding *pyrF*-based counter selection marker in *C. thermocellum*, where the continued presence of the mutant *pyrF* allele causes a growth defect in the absence of exogenous uracil [19]. The *C. acetobutylicum pyrE* deletion strain is unable to grow at all in CMBS media, but grows at a rate that is indistinguishable from the wild-type when the media is supplemented with exogenous uracil. The mutant produces essentially the same levels of solvents as the wild-type with possibly a slight reduction in butanol levels (Additional file 4: Fig. S5). However, any reduction is of no consequence, as the *pyrE* mutation is always repaired to prototrophy, using the ACE correction vector pMTL-ME6, with restoration of wild-type solvent yields (Additional file 4: Fig. S5). More importantly, mutants created in *pyrE* strains (by whatever means) can be rapidly complemented using ACE vectors by the introduction of a functional copy of the inactivated gene concomitant with restoration of the *pyrE* allele to wild-type. The extra effort involved in the deployment of ACE vectors compared to the use of automomous complementation vectors is minimal. They require the same amount of effort in terms of construction and transfer into the desired bacterial host. Transformants carrying autonomous complementation vectors are purified by restreaking, whereas a transformant carrying an ACE complementation vector first needs to be restreaked onto a minimal plate lacking uracil, before purification by re-streaking of the selected prototrophic cell lines. The extra effort, therefore, equates to the time it takes for a uracil prototrophic colonies to develop, ca. 2 days in the case of *C. acetobutylicum.* The efficiency of ACE is such that success is assured and, moreover, cannot suffer from false positives as the nature of the *pyrE* deletion is such that reversion of the mutation is impossible. Although the effort required for ACE-mediated complementation is minimal, the benefits are considerable. It avoids the phenotypic effects frequently observed with high copy number plasmids and dispenses with the need to add antibiotic to ensure the retention of the complementing plasmid. Such antibiotic addition can affect phenotype and necessitate the inclusion in any phenotypic assessments of the mutant a vector only control.

The benefits of the presence of the *pyrE* locus are such, that there is a rational argument for using *pyrE* mutant hosts, and their cognate ACE correction vectors (e.g., pMTL-ME6, pMTL-ME6C and pMTL-ME6X), with any particular mutagen, including the ClosTron and any of the available negative selection markers [18–23]. Moreover, the *pyrE* allele represents an ideal position where other application-specific modules may be inserted, such as a sigma factor to allow deployment of a *mariner* transposon [37], hydrolases [38] and therapeutic genes in cancer delivery vehicles [39]. In view of these uses, it is important that one establishes that no detrimental SNVs or Indels have arisen in the genome during the construction of the *pyrE* host. In the case of the previously described *C. difficile pyrE* hosts, no such alterations were noted [25]. In this study a single G > A substitution (Arg > Lys) was evident in the *pyrE* mutant in a gene (CA_C3553) annotated as a LacI family transcriptional regulator. Whilst significance of this change is unknown, its existence is immaterial since the *pyrE* mutant was subsequently remade in the re-acquired ATCC 824 strain during which no extra SNVs or Indels arose. This strain (CRG3899) is, therefore, the ATCC 824 host of choice for mutational work in *C. acetobutylicum*.

The number of SNVs and Indels in the ATCC strain obtained from the University of Rostock, originally as the strain to be used by all consortium members of the ERANET SysMO project COSMIC, was surprising. However, upon resequencing it was apparent that the bulk of the changes were also present in the 824 deposit that currently resides at the ATCC. As a consequence it seems likely that these 175 SNVs and 48 Indels represent mistakes in the original genome sequence, the first clostridial genome to be determined and, therefore, at a time when technologies were less sophisticated [30]. Their authenticity seems reasonably certain given that CLC Bio Workbench called these differences in four independent genome sequence runs with the DNA of the current ATCC 824, the COSMIC consortium strain and the *pyrE* mutants of both these strains. Moreover, in every case, correction of the identified synonymous SNVs and Indels in the ATC 824 NC_003030 and NC_001988 sequences that resided in coding regions resulted in ORFs that shared 100 % identity with the equivalent encoded proteins in the genome of *C. acetobutylicum* DSM 1731. Prior to this correction, some 90 of the ATC 824 encoded proteins were not 100 % identical to their DSM 1731 counterparts and in a number of instances either initiated at a different point in the genome (three) or where substantially foreshortened (twelve). Following correction of the latter changes, the C-terminal sequences of the encoding proteins were extended to give full-length sequences that were identical to the DSM 1731 homologues. In a number of instances (seven) the changes resulted in the fusion of two formerly distinct genes in the ATCC genome (NC_003030) to give a single CDS identical to that present in DSM 1731 (NC_015687). The full list of changes is given in the Additional file 2: Table S2.

A number of sequences were, however, unique to the COSMIC consortium strain. These equated to 2 SNVs and 1 deletion. One of the SNVs occurs in the coding region of CA_C1534 (C to A, position 1678694) causing an Ala to Ser substitution in an ABC-type iron (III) transport system (ATPase component) at position 303. The second SNV is a G to A substitution at position 3241095 just upstream of CA_C3087 (phosphoenolpyruvate-protein kinase). This particular substitution resides just upstream of the predicted -10 region of the promoter of CA_C3087 within the -35 region and could conceivably effect promoter activity. All of these SNVs were confirmed as present in the COSMIC strain, and not the re-sourced ATCC 824 strain, by Sanger sequencing of appropriately PCR amplified fragments. More significant was the 4916-nt deletion within the pSOL1 megaplasmid (position 69715 to 74631, NC_001988) that encompassed a putative mannose PTS and related genes (Table 3). The presence of this deletion in the COSMIC consortium strain, but not the freshly sourced ATCC 824 strain, was confirmed by the PCR and subsequent sequencing of the amplified fragment using appropriate primers. A low number of reads across the deleted region were, however, present in the Illumina sequencing run suggesting that the strain represented a mixed population, in which the majority of the cells harboured a copy of the pSOL1 plasmid carrying the ca. 5 kb deletion. As those cells carrying the deletion appeared to be in the majority, it was not unsurprising that the selected *pyrE* mutant was derived from this dominant subpopulation.

**Fig. 7:**
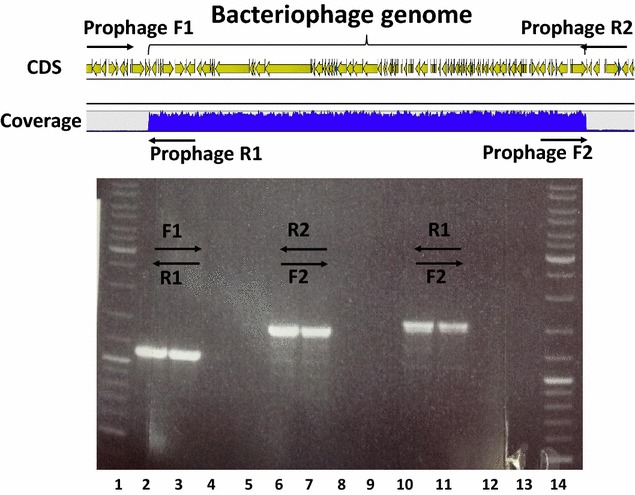
Evidence for excision of a prophage from the genome of *C. acetobutylicum* ATCC strain COSMIC. Screen shot of the mapped paired end reads of ATCC 824 COSMIC against the NC_003030 reference sequence over the region 2016385-2069765 encompassing a prophage. The indicated primers were used to show the region was present both in the chromosome (primers prophage-F1 and prophage-R1 and prophage-F2 and prophage-R2) and as an excised circular form (primers prophage-R1 and prophage-F2). The coverage for the genome was on average just over 200, whereas for the prophage encoding region it was over 4000

During the mapping of Illumina reads derived from the ATCC 824 COSMIC strain and its *pyrE* mutant, but not the re-acquired ATCC 824 strain, it was noted that read coverage over a ca. 53 kb region from position 2016385-2069765 (in NC_003030) was some 20-fold higher (over 4000 reads) than the rest of the genome (around 200 reads) (Fig. 7). This region initiates with CA_C1867, annotated as a phage-related, Xre family transcription regulator, and ends with CA_C1957 which encodes a site specific recombinase. In between are 89 CDS encoding proteins with varying degrees of homology to proteins normally associated with bacteriophages. It suggests that during growth of the organism to prepare the DNA, the prophage most likely left the lysogenic state. Indeed, through the design of appropriate primers, it was possible to show, at least in some of the population from which the DNA was prepared, that the region had become circularised (Fig. 7). A similar increased coverage was not seen in the DNA that was prepared from the re-acquired ATCC 824.

It has previously been reported [40] that the DSM 1731 genome contains 345 single nucleotide variations (SNVs) between its chromosome and that of ATCC 824. Based on the revision to the ATCC 824 genome sequence proposed here, this would be expected to reduce that number to 170. However, mapping of the ATCC 824, paired-end Illumina reads generated as part of this study to the DSM 1731 reference sequence NC_015687 suggests that the actual number of SNVs is 27, with a further two deletions and two insertions, one of which is the previously reported 1753 bp insertion at position 1278525 (see Additional file 3: Table S3 for the full list). In the case of the megaplasmid of ATCC 824, pSOL1 (Table 3), our re-sequence analysis identified a total of 4 SNVs and 1 deletion that were different to the published sequence (NC_001988). All four SNVs and the single deletion were present in the megaplasmid of DSM 1731, pSMBa, suggesting that the original sequence of pSOL1 was incorrect. The deletion identified was a 4-bp deletion (ACTC) at position 191997 previously reported to distinguish pSMBa, and the megaplasmid in *C. acetobutylicum* EA 2018, from pSOL1 [40]. All five changes were confirmed as correct by Sanger sequencing.

Having identified errors in both the published chromosomal (NC_003030) and pSOL1 (NC_001988) sequences, we compiled a new annotated reference sequence of both (Additional files 5, 6, respectively) that contained the corrected chromosomal and pSOL1 sequences (Additional file 5, 6). These sequences were then used as references for the various Illumina sequence reads generated during this project. Using CRG_003030/CRG_001988, no SNVs or Indels were called during the analysis of the reads generated from the re-acquired ATCC 824 genome, and only those expected were called when sequence reads from the two *pyrE* mutants and the ATCC 824 COSMIC strain were used. Coincidentally, a new SNV was identified in the COSMIC strain in CA_C1989 which was not originally identified as it agreed with the incorrect NC_003030 sequence at position 2102286. Thus, the substitution of a ‘A’ for ‘C’ at CA_C1989 position 111 resulted in replacement of a Gln with a Pro residue and an encoded Iron ABC transporter ATP-binding protein that is no longer identical to SMB_G2021 of DSM 1731. In addition, two SNVs were shown to be present in pSOL1 compared to pSMBa, resulting in amino acid substitutions in two germination proteins (Table 3). These were at position 82 of an amino acid permease, Grkb1 (Gly in pSMBa, Val in pSOL1) and at position 407 of a germination receptor, GerA (Ala in pSMB, Val in pSOL1).

## Conclusions

The data presented here have demonstrated that in-frame deletion, marker-less mutants can be isolated in the biobutanol organism, *C. acetobutylicum,* through a two-stage process of single crossover integration and the subsequent isolation of double crossover excision events using replication-defective plasmids and either the counter selection marker *codA* or *pyrE.* Either marker appears equally effective, but there is considerable merit in using a *pyrE* mutant as the host because, through the use of ACE vectors, mutants created in such strains (by whatever means) can be rapidly complemented. This avoids the phenotypic effects frequently observed with high copy number plasmids and dispenses with the need to add antibiotic to ensure the retention of the complementing plasmid. Our study has also revealed a surprising number of errors in the the original genome sequence of ATCC 824, while at the same time emphasising the need to re-sequence commonly used laboratory strains.

## Methods

### Bacterial strains and routine culture conditions

Bacterial strains and plasmids used in this study are detailed in Table 1. *Escherichia coli* TOP10 (Invitrogen) was cultured aerobically (37 °C; shaking at 200 rpm) in Luria–Bertani LB medium supplemented with chloramphenicol (25 g/ml) where appropriate for plasmid cloning and storage, and TOP10 containing pAN2 plasmid [13] was used for in vivo methylation of plasmid DNA prior to transformation of *C. acetobutylicum* ATCC 824. *C. acetobutylicum* ATCC 824 was routinely grown anaerobically at 37 °C under an atmosphere of N2:H2:CO2 (80:10:10, vol:vol:vol) in an anaerobic workstation (Don Whitley, Yorkshire, UK.) using media pre-reduced overnight under the same conditions and grown in 2xYTG pH5.2 broth [41], *Clostridium* Basal Medium (CBM) [42] or on Clostridial Growth Medium (CGM) agar [43] supplemented with Tm (15 µg/ml) where appropriate. Uracil was supplemented at 20 µg/ml or 5-Fluoroorotic acid (FOA) (Europa Bioproducts Ltd) at 400 µg/ml in the media in the case of *C. acetobutylicum* ATCC 824 *pyrE* mutant. All reagents, unless noted, were purchased from Sigma-Aldrich (Tables 2, 3).

**Table 1:** Strains and plasmids used in this study

Strain/plasmid	Relevant features	Reference/source
Strains		
*E. coli* Top10	F- *mcrA Δ(mrr*-*hsdRMS*-*mcrBC) Φ80lacZΔM15 ΔlacX74 deoR recA1 araD139 Δ(ara*-*leu)7697 galU galK rpsL* (Str^R^) *endA1 nupG*	Invitrogen
*C. acetobutylicum* ATCC 824 (COSMIC)	CRG1268 strain used in the ERANET (http://www.sysmo.net) SysMO project COSMIC. Originally from G. Bennet, RICE University, USA	H Bahl University of Rostock
*C. acetobutylicum* ATCC 824	CRG3286, resourced from the ATCC in November 2011	ATCC
*C. acetobutylicum* CRG1545	*ΔpyrE* mutant of CRG1268 made using the ACE vector pMTL-JH12	[24]
*C. acetobutylicum* CRG3899	*ΔpyrE* mutant of CRG3286 equivalent to CRG1268	This study
*C. acetobutylicum* CRG3520	Δ*spo0A* of CRG1545 (Δ*pyrE*) made using pMTL-ME3::*spo0A*	This study
*C. acetobutylicum* CRG4889	Δ*spo0A* mutant of CRG1545 generated from CRG3520 by restoration of the *pyrE* allele to wild-type using pMTL-ME6	This study
*C. acetobutylicum* CRG4890	CRG3520 (Δ*spo0A*) carrying a functional copy of *spo0A* and its own promoter inserted into the genome using the ACE vector pMTL-ME6X::*spo0A* concomitant with restoration of the *pyrE* allele	This study
*C. acetobutylicum* CRG4891	CRG3520 (Δ*spo0A*) carrying a functional copy of *spo0A* under the control of the *fdx* promoter inserted into the genome using the ACE vector pMTL-ME6X::*spo0A* concomitant with restoration of *pyrE*	This study
*C. acetobutylicum* CRG4893	In-frame deletion mutant of *amyP* (CaP0168) made using pMTL-ME3::*amyP* (*pyrE* vector with *amyP* KO cassette)	This study
*C. acetobutylicum* CRG4894	restoration of the *pyrE* allele to wild-type using pMTL-ME6	This study
*C. acetobutylicum* CRG4896	CRG4893 (Δ*amyP*) carrying a functional copy of *amyP* and its own promoter inserted into the genome using the ACE vector pMTL-ME6C::*amyP* concomitant with restoration of the *pyrE* allele	This study
*C. acetobutylicum* CRG4897	CRG4894 (Δ*amyP*) carrying a functional copy of *amyP* under the control of the *fdx* promoter inserted into the genome using the ACE vector pMTL-ME6X::*amyP* concomitant with restoration of *pyrE* allele	This study
*C. acetobutylicum* CRG4883	In-frame deletion mutant of *glgA* (Cac2239) made using pMTL-ME3::*glgA* (*pyrE* vector with *glgA* KO cassette)	This study
*C. acetobutylicum* CRG4898	Δ*glgA* mutant of CRG4883 generated from CRG1545 by restoration of the *pyrE* allele to wild-type using pMTL-ME6	This study
*C. acetobutylicum* CRG4901	CRG4883 (Δ*glgA*) carrying a functional copy of *glgA* and its own promoter inserted into the genome using the ACE vector pMTL-ME6C::*glgA* concomitant with restoration of the *pyrE* allele	This study
*C. acetobutylicum* CRG4902	CRG4883 (Δ*glgA*) carrying a functional copy of *glgA* under the control of the *fdx* promoter inserted into the genome using the ACE vector pMTL-ME6X::*glgA* concomitant with restoration of *pyrE* allele	This study
*C. acetobutylicum* CRG2404	In-frame deletion mutant of *spo0A* gene made using pMTL-SC7515::*spo0A*	This study
*C. acetobutylicum* CRG2403	In-frame deletion mutant of the type II Restriction system Cac1502 made using pMTL-SC7515::*cac824I*	This study
Plasmids		
pAN-2	*E.coli* vector (ϕ3T I methyltransferase gene, *tet,* p15a)	CRG685 [13]
pMTL85141	*E.coli*- *Clostridium* Shuttle vector (pIM13 *catP* ColEI)	CRG1000 [31]
pMTL-ME2	*Clostridium* KO vector (pCB102 *catP* ColE1 *pyrE*)	CRG1668 [25]
pMTL-ME3	*Clostridium* KO vector (pIM13 *catP* ColE1 *pyrE*)	CRG1671This study
pMTLSC7315	*Clostridium* KO vector (pCB102 *catP* ColE1 *traJ codA*)	CRG1424 [21]
pMTLSC7515	*Clostridium* KO vector (pIM13 *catP* ColE1 *traJ codA*)	CRG1303 This study
pMTL-ME6	*C. acetobutylicum* ACE correction vector (pIM13 *catP* ColE1 `*pyrE*)	CRG1861 This study
pMTL-ME6C	*C. acetobutylicum* ACE complementation vector (pIM13 *catP* ColE1 *pyrE lacZ’* MCS)	CRG2483 [25]
pMTL-ME6X	*C. acetobutylicum* ACE expression vector (pIM13 *catP* ColE1 `*pyrE P*_*fdx,*_ *lacZ’* MCS)	CRG2482 [25]
pMTL-ME3::*spo0A*	pMTL-ME3 + *spo0A* KO cassette (pIM13 *catP* ColE1 *pyrE*)	CRG1701 This study
pMTL-ME3::*amyP*	pMTL-ME3 + *amyP* KO cassette (pIM13 *catP* ColE1 *pyrE*)	CRG3727 This study
pMTL-ME3::*glgA*	pMTL-ME3 + *glgA* KO cassette (pIM13 *catP* ColE1 *pyrE*)	CRG4881 This study
pMTL-SC7515::*spo0A*	pMTLSC7515 + *spo0A* KO cassette (pIM13 *catP* ColE1 *codA*)	CRG1672 This study
pMTL-SC7515::*cac824I*	pMTLSC7515 + *cac824I* KO cassette (pIM13 *catP* ColE1 *codA*)	CRG2293 This study
pMTL-ME6C::*spo0A*	ACE complementation vector for *spo0A*	CRG3739 This study
pMTL-ME6X::*spo0A*	ACE overexpression vector for *spo0A*	CRG3738 This study
pMTL-ME6C::*amyP*	ACE complementation vector for *amyP*	CRG3723 This study
pMTL-ME6X::*amyP*	ACE overexpression vector for *amyP*	CRG3722 This study
pMTL-ME6C::*glgA*	ACE complementation vector for *glgA*	CRG4900 This study
pMTL-ME6X::*glgA*	ACE overexpression vector for *glgA*	CRG4880 This study

**Table 2:** Unique SNVs and Indels, compared to the current ATCC 824 strain, in the laboratory ATCC 824 strain COSMIC and their *pyrE* mutants

Strain	NC_003030	Type	Ref	Allele	Feature	Change	AA Change	Function	CRG_003030^b^
ATCC 824 COSMIC	1678711	SNV	C	A	CA_C1534	907G > T	Ala303Ser	Iron ABC transporter ATP-binding protein	1678691
	2102314^a^	SNV	G(T)	G	CA_C1989	332A > C	Gln111Pro	Iron (III) ABC transporter ATPase	2102286
	3241117	SNV	G	T	Miscellaneous	–	–	Promoter region of CA_C3087	3241092
ATCC 824 COSMIC PyrE	3749367	SNV	G	A	CA_C3553	248G > A	Arg83Lys	LacI family transcriptional regulator	3749536

^a^sequence identical to CA_C1989 in NC_003030 annotation at position 2102314 (G), but once the sequence is corrected to a ‘T’, the COSMIC ‘G’ at this position becomes a SNV

^b^CRG_003030.gbk is a corrected annotation of the ATTC 824 chromosomal genome sequence and is available as a Additional file 5

**Table 3:** SNVs in the pSOL1 megaplasmid of *C. acetobutylicum* ATCC 824 COSMIC strain and re-acquired ATCC 824 strain

NC_001988	Type	Ref	Allele	Feature	Change	AA Change	Function/comment
ATCC 824 isolate re-acquired from the ATCC in November 2011							
18758	SNV	G	T	CA_P0017	11C > A	Ala4Glu	GrkB germination protein, 4Glu also in SMB_P016 of DSM 1731
54266	SNV	A	G	CA_P0053	317T > C	Leu106Pro	Xylanase XYNB, now identical to SMB_P052 of DSM 1731
56219	SNV	A	G	CA_P0055	506A > G	Asn169Ser	Hypothetical protein, now identical to SMB_P054 of DSM 1731
60555	SNV	G	T	CA_P0059	754C > A	His252Asn	Alcohol dehydrogenase, now identical to SMB_P058 of DSM 1731
191997	Del	ACTC	–	Deletion	–	–	Same deletion as in pSMBa of DSM1731
ATCC 824 COSMIC strain							
18758	SNV	G	T	CA_P0017	11C > A	Ala4Glu	GrkB germination protein, 4Glu also in SMB_P016 of DSM 1731
54266	SNV	A	G	CA_P0053	317T > C	Leu106Pro	Xylanase XYNB, now identical to SMB_P052 of DSM 1731
56219	SNV	A	G	CA_P0055	506A > G	Asn169Ser	Hypothetical protein, now identical to SMB_P054 of DSM 1731
60555	SNV	G	T	CA_P0059	754C > A	His252Asn	Alcohol dehydrogenase, now identical to SMB_P058 of DSM 1731
69715	Del	69715…74631		CA_P0066-71	del4916 bp	–	Unique to pSOL1, affects ptna, manY,ptnd, CA_P0069, CA_P0070, CA_p0071
191997	Del	ACTC	–	Deletion	–	–	Same deletion as in pSMBa of DSM1731
DSM 1731 (map positions are to NC_015686)							
18524	SNV	C	A	SMB_P016	245G > T	Gly82Val	GrkB, germination protein, Gly at position 82 instead of Val in CA_P0017
20626	SNV	G	A	SMB_P019	220C > T	Ala407Val	GrkA, germination receptor, Ala at position 407 instead of Val in CA_P0020

### Transformation

Chemically competent cells of *Escherichia coli* TOP10 were prepared using 0.1 M MgCl_2_ and 0.1 M CaCl_2_ as described in [43]. Ligation mixture or 10–20 µl or plasmids 1–2 µl were mixed with 50 µl of chemical competent cells and incubated on ice for 10–15 min and transformed by heat shock method at 42 °C for 1 min in a PCR block, incubated for 1 min on ice followed by addition of 1 ml LB and incubating at 37 °C, 200 rpm shaking for 30–60 min. Cells were centrifuged at full speed for 1 min and after re-suspending pellet in 200 µl of LB, 100 µl of cells was plated onto LB agar supplemented with chloramphenicol (25 g/ml) and incubated overnight at 37 °C. *C. acetobutylicum* ATCC 824 was transformed by electroporation as described by [31]. After transformation, cells were mixed in 10 ml of 2xYTG pH 5.2 and incubated at 37 °C for 2–3 h (supplemented with 20 µg/ml uracil in the case of *pyrE* mutant) followed by centrifugation at 5000 rpm at room temperature and re-suspending the pellet in 300 µl of 2xYTG pH 5.2 and plating 100 µl onto CGM agar supplemented with 15 µg/ml Tm and incubated at 37 °C in anaerobic conditions for 24–48 h.

### Cloning and PCR analysis

*C. acetobutylicum* ATCC 824 and *C. sporogenes* ATCC 15579 genomic DNAs for use in cloning and PCR analysis were prepared using QIAGEN DNeasy Blood and Tissue Kit in accordance with the manufacturer’s instructions and recommended pretreatment for Gram-positive bacteria using Lysozyme from chicken egg white (Cat L6876 Sigma-Aldrich) 20 ml/ml phosphate buffer saline (PBS) [25]. Screening PCRs were performed using DreamTaq™ green PCR master mix (Fisher Scientific UK Ltd) and Failsafe™ PCR system (Epicentre) for downstream cloning in accordance with the manufacturer’s instructions. Primers were designed using web tool available at http://primer3.ut.ee and are listed in Additional file 1: Table S1. All the DNA manipulations including restriction digestion, de-phosphorylation of 5′ end and ligation were carried out according to standard procedures as described in [44]. DNA extraction and purification from agarose gel and PCR reaction mixtures were undertaken using QIAquick Gel Extraction and PCR Purification Kit (Qiagen Ltd UK), respectively, and used according to the manufacturer’s instructions.

### Vector construction

The *C. difficile* knock-out (KO) vectors pMTL-YN3/pMTL-ME2 (*pyrE*) and pMTL-SC7315 (*codA*) are based on the pCB102 plasmid replication region [21]. As plasmids that utilise the pIM13 replicon have proven highly effective as integration vectors in *C. acetobutylicum,* the pCB102 replication regions of pMTL-ME2 and pMTL-SC7315 were replaced with that of pIM13. Both pMTL-ME2 and pMTL-SC7315 correspond to the pMTL80000 modular format [31]. Their Gram-positive replicons are, therefore, flanked by AscI and FseI restriction sites. Accordingly, their pCB102 replicons were excised by cleavage with AscI and FseI and replaced with 878 bp AscI/FseI fragment from the modular plasmid pMTL85141 [31] that encompassed the pIM13 replication region. The two new vectors were designated pMTL-ME3 (derived from pMTL-ME2) and pMTL-SC7515 (derived from pMTL-SC7315). For chromosomal complementation and overexpression at the *pyrE* locus in mutants made using pMTL-ME3, plasmids pMTL-ME6C and pMTL-ME6X were used, respectively [25]. For the ACE-mediated correction of the *pyrE* locus of CRG1545 back to wild-type, pMTL-ME6 a functional equivalent to pMTL-YN1 was made [25]. A fragment of 1850 bp, encompassing regions of *pyrE* and *hydA* genes, was cloned by PCR using primers *pyrE*-LHAv1.0-F1 and hydA-RHAv1.0-R2 from *C. acetobutylicum* genomic DNA and was digested with HindIII and BglII and a 939-bp fragment was purified from agarose gel and ligated into pMTL-JH14 [24] linearized with the same restriction enzymes and confirmed by restriction analysis and Sanger sequencing.

### KO plasmids

Allele exchange cassettes specific to target loci were either constructed by SOE PCR [45] or commercially synthesised. In every case the authenticity of each final plasmid was verified by restriction digestion and nucleotide sequencing. Each cassette was composed of a left-homology arm (LHA) and a right-homology arm (RHA), each 750 bp in size. The *spo0A* KO cassette was designed to make an in-frame deletion that removed 435 bp out of 846 bp and was composed of the *C. acetobutylicum* bases 2171926 to 2172675 (LHA) fused to bases 2173111 to 2173860 (RHA) by SOR PCR. The flanking primers used added GTTTAAAC (a PmeI recognition sequence) at each extremity allowing the 1500-bp fragment to be blunt-end cloned into TA cloning vector pCR2.1 3929 bp and subsequently re-isolated as a BamHI/NotI fragment and inserted between the BamHI and NotI sites of pMTL-ME3 and pMTL-SC7515 to yield plasmid pMTL-ME3::*spo0A* and pMTLSC7515::*spo0A*, respectively. The KO cassette for CA_P0168 (*amyP*) was designed to delete 2277 bp of the 2283 bp long gene and comprised the *C. acetobutylicum* bases 180839 to 181588 (LHA) fused to bases 183866 to 184615 (RHA). It was synthesised by Eurofine www.eurofinsgenomics.eu and was flanked by BamHI and NcoI restriction sites. Following cleavage by BamHI and NcoI it was inserted between the equivalent restriction sites of pMTL-ME3 to give pMTL-ME3::*amyP*. The KO cassette targeting the *glgA* gene was composed of bases 2332450 to 2333199 (LHA) of the *C. acetobutylicum* ATCC 824 genome fused bases 2334628 to 2335377 (RHA) and was designed to remove 1428 bp of the 1434 bp long gene. The PCR primers used were designed to position a NotI restriction recognition site adjacent to the LHA and a PmeI site at the extremity of the RHA. The cassette was isolated as a 1500-bp NotI/PmeI fragment and inserted between the NotI site and NheI (blunt-ended with T4 polymerase) sites of pMTL-ME3 to yield the KO plasmid pMTL-ME3::*glgA*. The in-frame deletion cassette for CA_C1502 (*cac824I*) consisted of the *C. acetobutylicum* bases 1645573 to 1646322 (LHA) fused to 1647031 to 1647780 (RHA), which removes 708 bp from the 714 bp long gene. PmeI restriction sites were introduced as part of the SOE PCR flanking primers in the LHA and RHA which allowed the 1500-bp fragment to be isolated following digestion with PmeI and inserted into the equivalent site of plasmid pMTLSC7515 to resultant in the KO plasmid pMTL-SC7515::*cac824I*.

### Complementation vectors

To complement those genes knocked out using the *pyrE* system, appropriate DNA fragments encompassing the structural gene and native promoter were cloned into the ACE vector pMTL-ME6C. The *spo0A* gene with its native promoter was amplified as a 1211-bp fragment using primers NotI-Cacspo0A-F1 and EcoRI-Cacspo0A-R1 and following appropriate digestion cloned between the NotI and EcoRI sites of pMTL-ME6C to result in the plasmid pMTL-ME6C::*spo0A*. Similarly, the *C. acetobutylicum* ATCC 824 *amyP* gene (CA_P0168) was PCR amplified using primers Pca_P-0168-F1 and Ca_P-0168-R1 and the 2505-bp PCR product cleaved with NotI and BamHI and inserted between the equivalent sites of pMTL-ME6C. The plasmid obtained was designated pMTL-ME6C::*amyP*. To bring about overexpression of the complementing gene, promoter-less copies were cloned into pMTL-ME6X downstream of the strong P_fdx_ promoter. The *spo0A* gene was PCR amplified from genomic DNA using primers NdeI-Cacspo0A-F1 and EcoRI-Cacspo0A-R1. The PCR product obtained (859 bp) was digested with NdeI and EcoRI and ligated downstream of the P_fdx_ promoter in pMTL-ME6X linearized with the same restriction enzymes, to make pMTL-ME6X::*spo0A*.

To construct an over-expression vector for *amyP*, the gene was PCR amplified from genomic DNA without its promoter using Ca_P-0168-F2 and Ca_P-0168-R1. The start codon of the gene was changed from TTG to ATG using AseI in the forward primer which was compatible with NdeI. The 2300-bp PCR product obtained was digested with AseI and BamHI and cloned in pMTL-ME6X linearized with NdeI and BamHI to make pMTL-ME6X::*amyP*. However, cells transformed with this plasmid were negative for amylase activity by the starch-iodine plate test. To remedy this lack of expression, the ATG was changed back to the original TTG start by making a quick change in the vector using oligonucleotides Qfdx-0168-F1 and Qfdx-0168-R1, while at the same time reducing the spacer region between the start codon and ribosome binding site by one base pair.

### Allelic exchange procedure

The procedure used for the isolation of the in-frame deletion mutants in *C. acetobutylicum* using either pMTL-ME3 or pMTL-SC7515 is essentially the same and based on the procedures described by Ng el et al. [25] and Cartman et al. [21], respectively. Derivatives of the two vectors carrying the required KO cassette were electroporated into either the wild-type (pMTL-SC7515) or the *pyrE* mutant strain (pMTL-ME3) and plated on CGM supplemented with 15 µg/ml Tm and incubated for 24–48 h. The largest representative colonies were selected and passaged twice by re-streaking onto the same agar media, at each stage choosing larger representative colonies. The cells of selected colonies were then screened by PCR to ascertain whether the isolated clonal population was a pure single crossover integrant, using appropriate pairs of primers. These comprised a forward primer complementary to the chromosome and a reverse primer specific to a plasmid encoded region. The presence of a PCR product indicated that the clones were single crossover integrants, and the size of PCR product indicated at which homology arm (LHA or RHA) recombination had happened. It was important to establish purity, as the presence of cells in which integration had not occurred would contribute to a high background level of colonies when subsequently plated on the counter selection agents, FOA and FC.

Pure single, crossover clones were plated onto CGM supplemented with 20 µg/ml uracil or un-supplemented CGM medium and incubated for 2–3 days to allow cells to undergo a second recombination event, resuspended in PBS and appropriate dilutions spread on CGM agar plates supplemented with either FOA (400 µg/ml) and uracil (1 µg/ml) or CBM supplemented with FC (100 µg/ml) for pMTL-ME3 and pMTL-SC7515, respectively, and incubated for 48 h. Only cells that have, respectively, lost *pyrE* or *codA* through excision of the plasmid and its subsequent loss from the cell may grow. The loss of the KO vector was confirmed by patch plating on CGM with or without Tm supplementation. As the excision event may be mediated by either of the two homology arms (LHA or RHA), the cell line that arises can be either mutant or wild-type. Where there is no homology arm preference, then the ratio of mutant to wild-type should be 50:50. However, this is rarely the case. Moreover, if one homology arm is favoured at the single crossover integration stage, then it will be favoured at the double crossover stage and as a consequence the proportion of wild-type derivatives will be higher.

Screening of FC^R^ and FOA^R^ clones by PCR was undertaken using primer pairs that flanked the desired deletion point and were complementary to chromosomally located sequence that resided external to the homology arms used. For example, Cac-spo0A-sF2 and Cac-spo0A-sR2 in the case of spo0A; Cac-1501-sF2 and Cac-1504-sR1 in the case of *cac824I*; Cac-amyP-sF2 and Cac-amyP-sR2 in the case of *amyP* and; Cac-glg-sF2 and Cac-glg-sR1 in the case of *glgA*. The PCR products generated were subjected to Sanger sequencing to confirm their authenticity.

### Correction of *pyrE* using ACE

The ACE procedure adopted is essentially as described before [24]. Plasmids pMTL-ME6 and the derivatives of pMTL-ME6C and pMTL-ME6X prepared from *E. coli* pAN2 were transformed into the appropriate *C. acetobutylicum* mutant and Tm^R^ transformants selected onto CGM agar supplemented with 15 µg/ml Tm and 20 µg/ml uracil. Selected large colonies (single crossover integrants) were then streaked onto CBM lacking uracil supplementation to select plasmid excised derivatives (double crossover cells) in which the *pyrE* allele had been restored to wild-type and the cells had become prototrophic. PCR primers Cac0026-sF2 and Cac-hydA-sR2 were used to screen for authentic clones and the amplified fragment subject to nucleotide sequencing.

### High-throughput sequencing

Phenol chloroform method was used to isolate genomic DNA from strains CRG1268 (COSMIC wild-type strain), CRG1545 (COSMIC strain *pyrE* mutant), CRG3286 (re-acquired ATCC 824) and CRG3899 (re-acquired ATCC 824 *pyrE* mutant). CRG1268, CRG1545 and CRG3286 were sequenced by Illumina hiseq (GATC, Germany) with read lengths of 101, 51 and 51, respectively. For the sequencing of CRG3899 paired-end libraries were prepared and sequenced (251 bp reads) using an Illumina MiSeq benchtop sequencer (Deepseq, University of Nottingham). Preparation of paired-end libraries as well as sequencing was performed as described by the manufacturers. Sequence reads were mapped to the reference sequences NC_003030 (chromosome) and NC_001988 (pSOL1) using CLC Genomics Workbench. We used a frequency of 90 % as a cut-off for SNV calling. Selected SNVs and InDels were confirmed by amplifying a few hundred base pairs up- and downstream of the area of interest and the amplicon was Sanger sequenced (Source BioScience, UK).

### Sporulation assay

Spore stocks of test strains were heat treated at 80 °C for 10 min before plating on pre-reduced CBM agar and incubation for 24 h. Triplicate pre-cultures were prepared by resuspending a loop-full of colonies from each strain into three universal tubes containing 10 ml CBMS medium (CBM + 0.5 % w/v CaCO_3_, 5 % w/v glucose) and incubated overnight. The following day, triplicate sporulation assay cultures (30 ml CBMS medium in 50 ml-Falcon tubes) were inoculated to an initial OD_600_ nm 0.048-0.0065 from the pre-cultures. Aliquots of 200 µl were removed at 0, 24, 48, 72, 96, and 120 h. Samples were heated at 80 °C for 10 min in a water bath and dilutions (10^−1^–10^−6^) were prepared in 96 well microtitre plate. From each strain, 20 µl volumes were spotted onto CBM agar and incubated for 48 h. The number of colonies was counted and heat_resistant Colony Forming Units per ml (CFU/ml) was calculated.

### Detection of granulose

Granulose accumulation was tested by growing the *C. acetobutylicum* ATCC 824 strains on agar-solidified CBM containing 5 % glucose to promote granulose accumulation. After incubation for 72 h, plates were exposed to iodine vapour, staining granulose-containing colonies dark brown.

## Additional files

10.1186/s13068-015-0410-0 This file contains all oligonucleotides used in this study.
10.1186/s13068-015-0410-0 This file contains full details of SNV and Indels of ATCC 824 against the GenBank ATCC 824 sequence, NC_003030.
10.1186/s13068-015-0410-0 This file contains full details of the SNVs and Indels of ATCC 824 Illumina reads against the DSM 1731 GenBank genome sequence, NC_015686.
10.1186/s13068-015-0410-0 Supplementary Figures S1, S2, S3 and S4.
10.1186/s13068-015-0410-0 Corrected annotated chromosomal genome sequence of ATCC 824.
10.1186/s13068-015-0410-0 Corrected annotated sequence of the pSOL1 megaplasmid of ATCC 824.

## Abbreviations

ACE: allele-Coupled Exchange
CBM: clostridial basal medium
CGM: clostridial growth medium
FC: 5-fluorocytosine
FOA: 5-fluorouracil
Indels: insertions and deletions
KO: knock-out
SNV: single nucleotide variations
SOE: splicing by overlap extension
Tm: thiamphenicol
