# Supplementary material for: Mutant generation by allelic exchange and genome resequencing of the biobutanol organism Clostridium acetobutylicum ATCC 824
Source: Biotechnol Biofuels. 2016 Jan 4;9:4. doi: 10.1186/s13068-015-0410-0 (PMC4700727; doi:10.1186/s13068-015-0410-0)
Supplement: Supplementary file 1 — 10.1186/s13068-015-0410-0 This file contains all oligonucleotides used in this study. [file 13068_2015_410_MOESM1_ESM.docx]

**Table S1. Oligonucleotides and primers used in this study**

**Name Sequence Reference/Source**

*lacZa*-sF2 ACTGGCCGTCGTTTTACAACGTCGTG [24]

Cac0026-sF2 TAGCACAATTGTATTTGGACTTCTTTAAATAAAAACATGG [24]

Cac-*hydA*-sR2 TTGATGATGTTTGTCTTGATGACTCAACATGC [24]

*pyrE*-LHAv1.0-F1 CCTGCAGGAGAGTAATGTACTTACCTTTGGGGATTTCATAAC [24]

*hydA*-RHAv1.0-R2 GGCGCGCCTGTTGCTGCTTTAAAAGAAAAATCCCATATAGAAAA

AGTTCAAGAAGC [24]

M13F GTAAAACGACGGCCAG Invitrogen Ltd

SC7-R AGATCCTTTGATCTTTTCTACGGGGTCTGACGCTCAGTGG [21]

SC7-F GACGGATTTCACATTTGCCGTTTTGTAAACGAATTGCAGG [21]

*Csp*-*pyrE*-HpaI-sF1 AATATTgttaacAATATTGTTAACTAAGGAGAAGATATAAATGAGTAA

TATAAATGTTATAGATATATTAAAAGAATCAAAT This study

*Csp-pyrE-HpaI-sR1* AATATTgttaacTTATTTTTGTTCTCTACTACCTGGTTTTACAAAAGGT This study

Cac-1501-sF2 GGATTTTTCTCATTTACCCCAGA This study

Cac-1504-sR1 GCTGTAGTGTAAACTTGTTCTTTG This study

Cac-*spo0A*-sF2 CTCACCCTTTCTTTCCATCAC This study

Cac-*spo0A*-sR2 CCTAAAAGTATGATGATTAAAGTTACAGATGC This study

NotI-Cac*spo0A*-F1 TAAgcggccgcAAATTGAGTTTATTAAGTATAACCCTATATATAGG This study

EcoRI-Cac*spo0A*-R1 TAATACgaattcTAACTTAGCTAACTTTATTTTTAAGTC This study

NdeI-Cac*spo0A*-F1 ATATcatatgGAAAGTAGAAAAATAAGTGTT This study

Pca_P0168-F1 AAAAgcggccgcAGATTAAAAATAATTGCAACAAGTGTGTTGAC This study

Ca_P0168-R1 TTTTggatccTTAATTATTTGTTGAAGAAAAATTATAGTTATTACTGT This study

Ca_P0168-F1 AAAAcccgggTTGAGTAAACGTTCTAAATTGTTAAAAAGAAG This study

Ca_P0168-F2 AAAAattaatGAGTAAACGTTCTAAATTGTTAAAAAGAAG This study

Qfdx-0168-F1 GTGTAATTTTTAAGGAGGTGTGTTACATTTGAGTAAACGTTCTAAAT

TGTTAAAAAG This study

Qfdx-0168-R1 CTTTTTAACAATTTAGAACGTTTACTCAAATGTAACACACCTCCTTAA

AAATTACAC This study

Cac-*amyP*-sF2 GCTCTGGCACAACTTTACGA This study

Cac-*amyP*-sR2 GCACCATTTTGTGATGTGAAA This study

ME3-sR2 GAGCGAAGCGAATAAGCGTC This study

ME3-sF2 ACCGTATTACCGCCTTTGAGTGAG This study

*glg*1-F1 ATATgcggccgcATACAAAAGAACCAATAATGGTAAACGA This study

*glg*3-SOE-R1 CTCCCTAATTAAAAAATTATAATTACAAATTGGTTTCCCCCTTTATTA This study

*glg*4-SOE-F1 TAATAAAGGGGGAAACCAATTTGTAATTATAATTTTTTAATTAGGGAGThis study

*glg*2-R1 ATATgtttaaacTTAGGTGTAAAAGCTATTGTATTGCTGC This study

Cac-*glg*-sF2 GAAGCACACAGGAGAAAGTAATCCTCTCCTC This study

Cac-*glg*-sR1 CCATATAAAATATATGAAGCCGTGTTAATAGC This study

Prophage F1 TATGTTGAGGGTGATGTG This study

Prophage R1 GTGAGGAAGGGAAAAGTG This study

Prophage F2 TGAACTATGGTTGGAAGTG This study

Prophagr R2 GTTGGGTAGGAAGATTGG This study
