# Supplementary material for: Mutant generation by allelic exchange and genome resequencing of the biobutanol organism Clostridium acetobutylicum ATCC 824
Source: Biotechnol Biofuels. 2016 Jan 4;9:4. doi: 10.1186/s13068-015-0410-0 (PMC4700727; doi:10.1186/s13068-015-0410-0)
Supplement: Supplementary file 4 — 10.1186/s13068-015-0410-0 Supplementary Figures S1, S2, S3 and S4. [file 13068_2015_410_MOESM4_ESM.docx]

**SUPPLEMENTARY FILES**

**Mutant generation by allelic exchange and genome resequencing of the biobutanol organism *Clostridium acetobutylicum* ATCC 824**

Muhammad Ehsaan, Wouter Kuit^+^, Ying Zhang, Stephen T. Cartman^#^, John T. Heap^$^, Klaus Winzer and Nigel P. Minton*


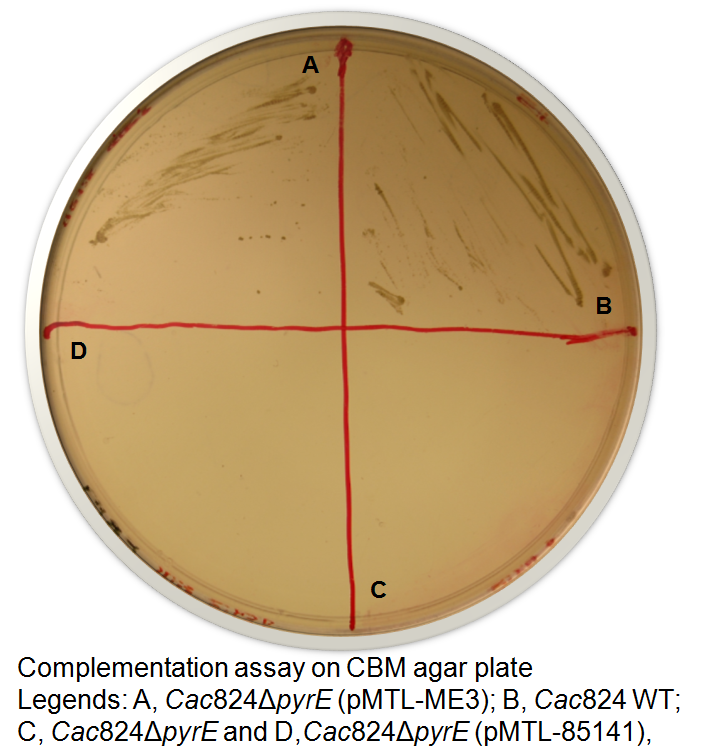


**Figure S1. Confirmation of functionality of the *C. sporogenes pyrE* gene in *C. acetobutylicum*.**

Cells of the *C. acetobutylicum* ATCC 824 COSMIC strain *pyrE* mutant (CRG1545), with and without various plasmids, were streaked onto CGM agar supplemented with 15 μg/ml Tm and 20 μg/ml uracil and incubated for 24 h. A, CRG1545 containing pMTL-ME3; B, *C. acetobutylicum* ATCC 824 COSMIC wild-type (CRG1268); C, plasmid-free CRG1545, and; D, CRG1545 containing the vector pMTL85141.


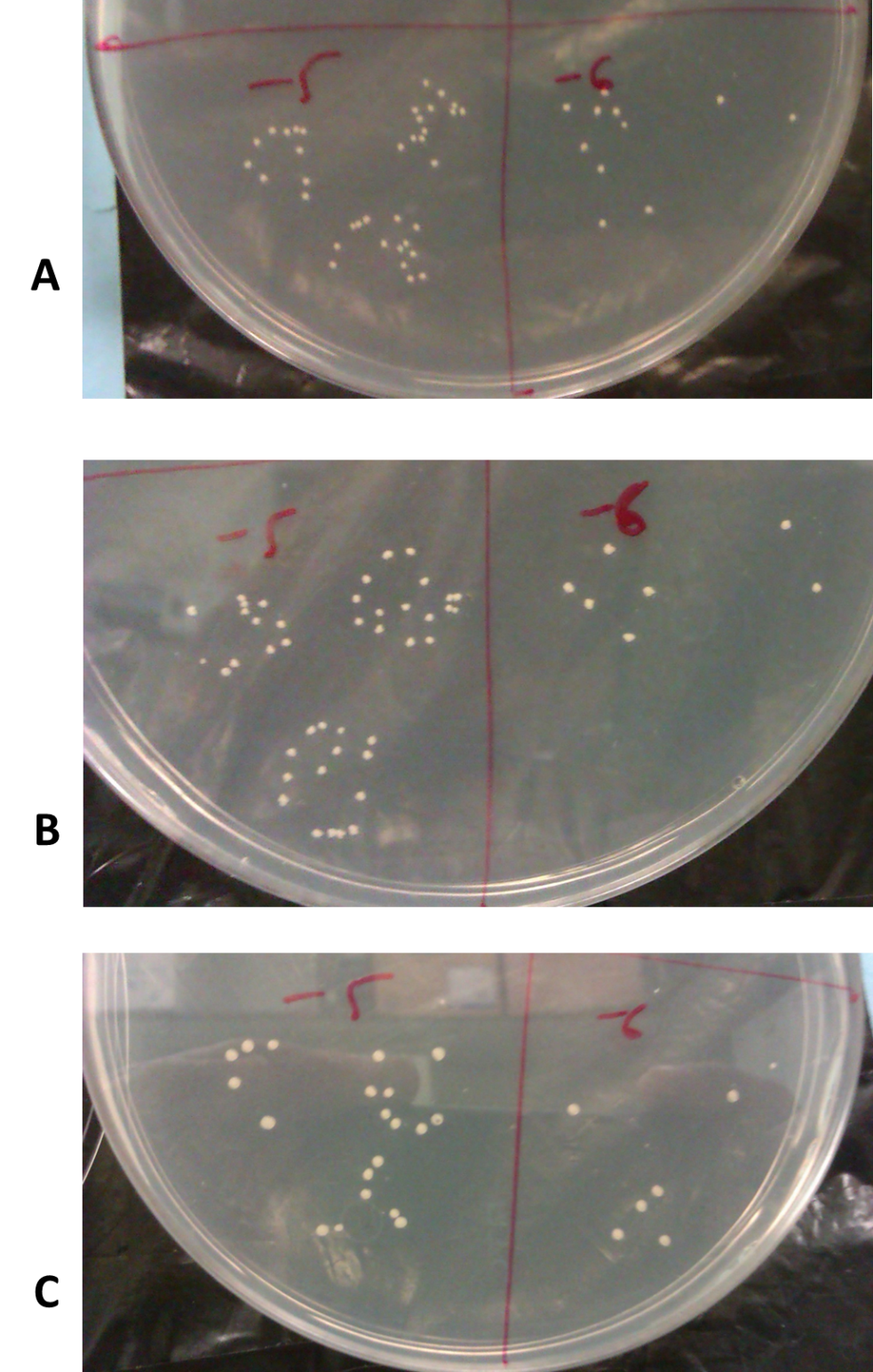


**Figure S2. Effect of over expression of *spo0A* on colony morphology of complemented mutants**

Cultures were grown in liquid CBM medium and 20 µl volume spotted on CBM agar and incubated for 24hr anaerobically at 37^o^C. Panel A: *C. acetobutylicum* ATCC 824 COSMIC strain wild-type. Panel B: *C. acetobutylicum* CRG4890 carrying a functional copy of *spo0A* and its own promoter inserted into the genome using the ACE vector pMTL-ME6C::*spo0A* concomitant with restoration of the *pyrE* allele. Panel C: *C. acetobutylicum* CRG4891 carrying a functional copy of *spo0A* under the control of the *fdx* promoter inserted into the genome using the ACE vector pMTL-ME6X::*spo0A* concomitant with restoration of *pyrE*.

| 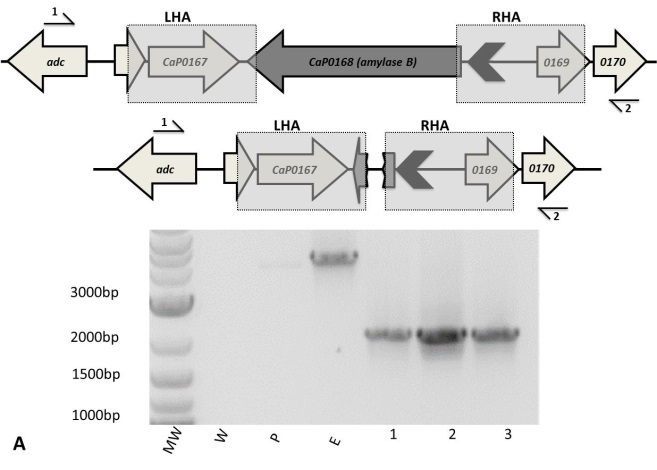 | 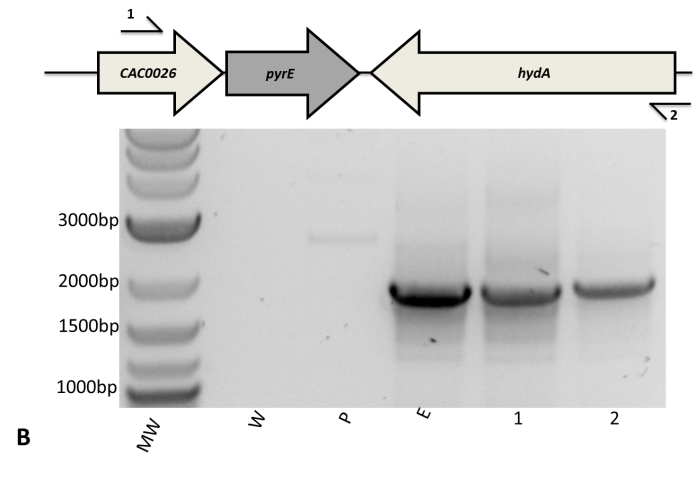 |
| --- | --- |
| 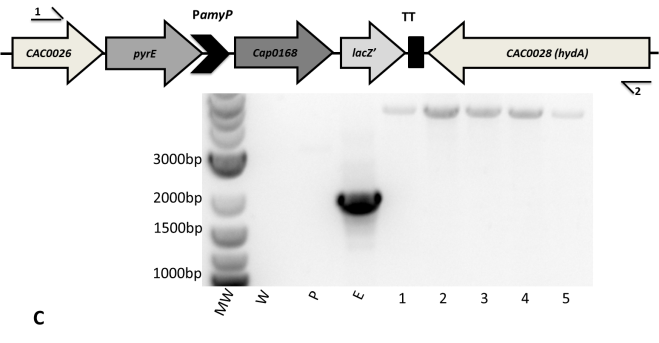 | 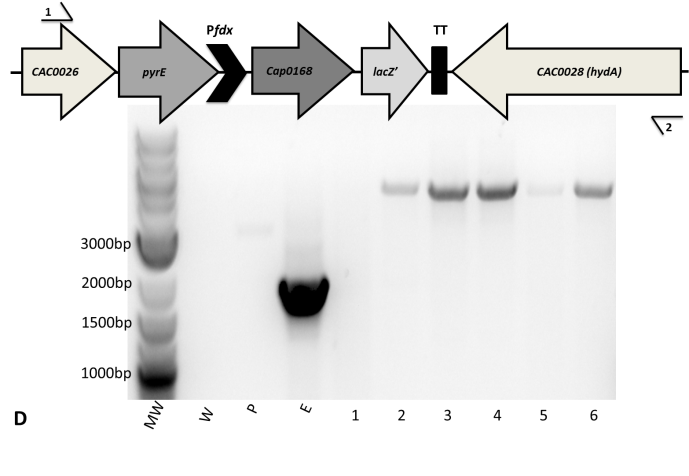 |

**Figure S3. Isolation of in-frame deletion mutants of *amyA***

Schematic representations of the wild-type and mutant genomes in the targeted regions are shown above each electrophoretogram, together with the relative position of the two primers used and the extent of the Left Homology Arm (LHA) and Right Homology Arm (RHA) employed to mediate recombination. In both panels: MW, 2-log DNA marker (NEB) molecular weight marker; ‘W’, is a PCR with no DNA; ‘P’, is a PCR using the plasmid only, and; ‘E’ is the *pyrE* minus parent strain CRG1545. Panel A. PCR screening of three FOA^R^ colonies (labelled lanes 1 to 3) using primers Cac-amyP-sF2 (1) and Cac-amyP-sR2 (2). Expected fragment sizes are 2078 bp in the deletion mutant and a 4355 bp fragment in the *pyrE* minus parent strain CRG1545, respectively. Accordingly, all three strains screened are mutants. Panel B. PCR screening of the two uracil prototroph clones using flanking primers Cac0026-sF2 (1) and Cac-hydA-sR2 (2). The expected PCR product for the *pyrE* mutant parent strain is 1936 bp and for the strain in which the *pyrE* allele has been restored to wild-type with pMTL-ME6 is 1989 bp. Panel C. PCR screening of five uracil prototroph using flanking primers Cac0026-sF2 (1) and Cac-hydA-sR2 (2). The expected PCR product for the parent *pyrE* minus strain is 1936 bp and for the strain in which the *pyrE* allele has been restored to wild-type with pMTL-ME6C-*amyP* is 5124 bp. Panel D. PCR screening of six uracil prototroph using flanking primers Cac0026-sF2 (1) and Cac-hydA-sR2 (2). The expected PCR product for the *pyrE* mutant parent strain is 1936 bp and for the strain in which the *pyrE* allele has been restored to wild-type with pMTL-ME6X::*amyP* is 5128 bp in clones 2-6 while in clone 1 PCR was unsuccessful.

| 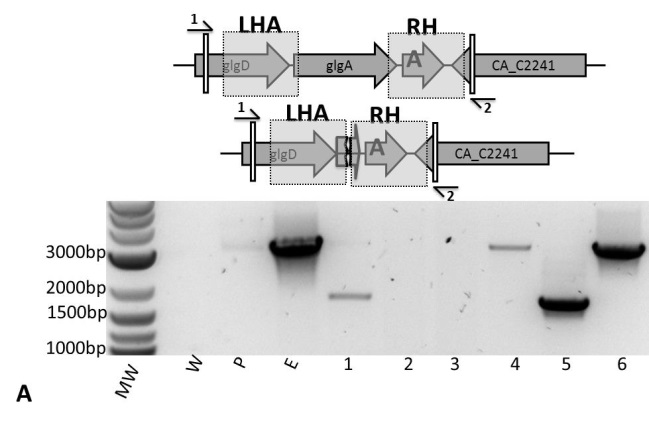 | 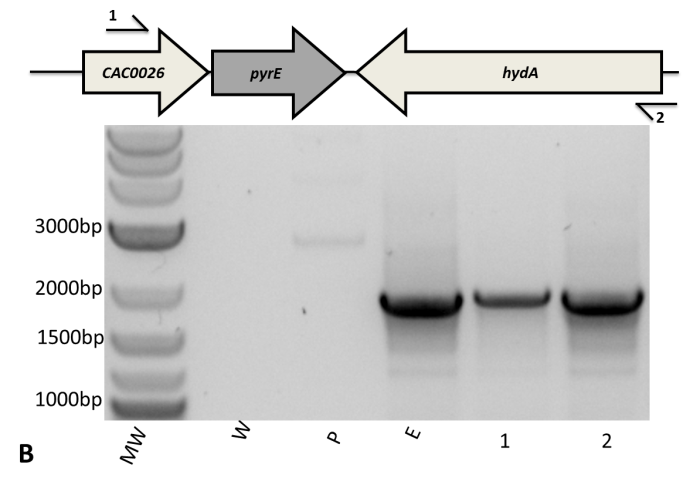 |
| --- | --- |
| 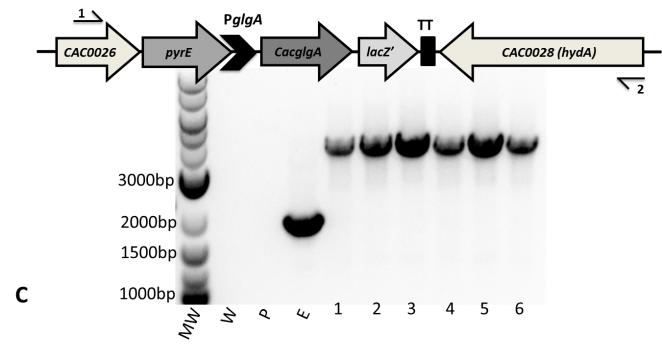 | 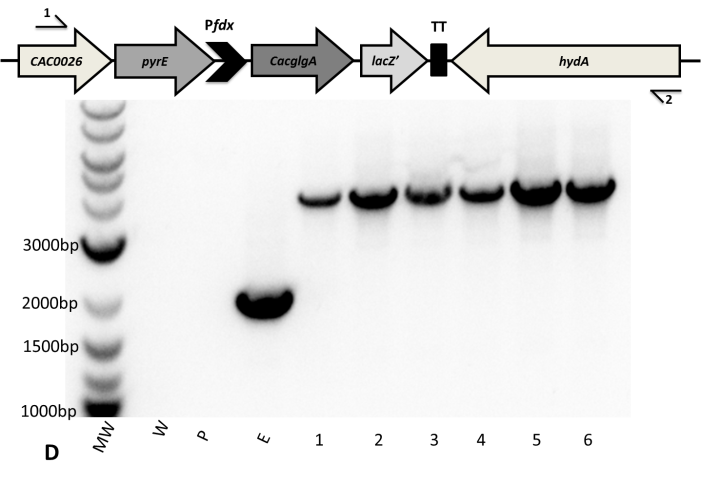 |

**Figure S4. Isolation of in-frame deletion mutants of glgA**

Schematic representations of the wild-type and mutant genomes in the targeted regions are shown above each electrophoretogram, together with the relative position of the two primers used and the extent of the Left Homology Arm (LHA) and Right Homology Arm (RHA) employed to mediate recombination. In both panels: MW, 2-log DNA marker (NEB) molecular weight marker; ‘W’, is a PCR with no DNA; ‘P’, is a PCR using the plasmid only, and; ‘E’ is the *pyrE* minus parent strain CRG1545. Panel A. PCR screening of six FOA^R^ colonies (labelled lanes 1 to 6) using primers Cac-glg-sF2 (1) and Cac-glg-sR1 (2). Expected fragment sizes are 1684 bp in the deletion mutant and a 3112 bp fragment in the *pyrE* minus parent strain CRG1545, respectively. Accordingly, labelled lanes 1 and 5 are mutants, lanes 4 and 6 are *pyrE* minus parent strain CRG1545. In lanes 2 and 3 the screening was unsuccesful. Panel B. PCR screening of the two uracil prototroph clones using flanking primers Cac0026-sF2 (1) and Cac-hydA-sR2 (2). The expected PCR product for the *pyrE* mutant parent strain is 1936 bp and for the strain in which the *pyrE* allele has been restored to wild-type with pMTL-ME6 is 1989 bp. Panel C. PCR screening of six uracil prototroph using flanking primers Cac0026-sF2 (1) and Cac-hydA-sR2 (2). The expected PCR product for the parent *pyrE* minus strain is 1936 bp and for the strain in which the *pyrE* allele has been restored to wild-type with pMTL-ME6C::*glgA* is 4292 bp. Panel D. PCR screening of six uracil prototroph using flanking primers Cac0026-sF2 (1) and Cac-hydA-sR2 (2). The expected PCR product for the *pyrE* mutant parent strain is 1936 bp and for the strain in which the *pyrE* allele has been restored to wild-type with pMTL-ME6X::*glgA*  is 4278 bp.


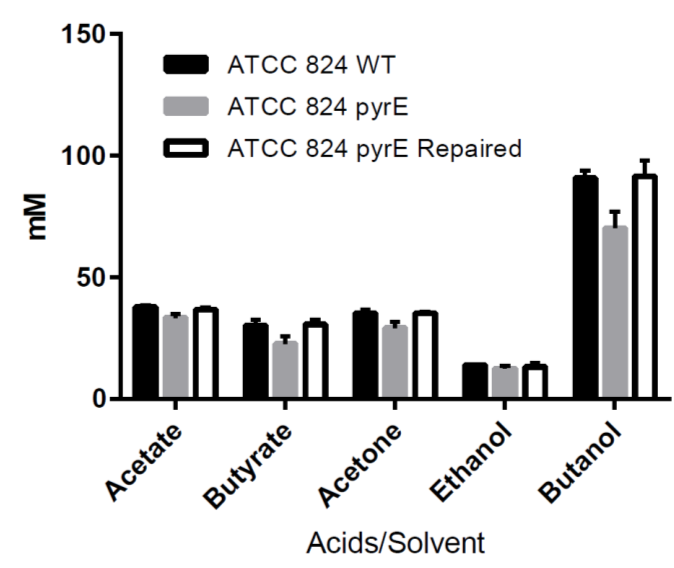


**Figure S5. Solvent profiles of the *pyrE* mutant compared to the wildtype and derivative restored to prototrophy using ACE.**

Strains were grown in batch culture using CBMS broth containing 5% glucose and 0.5% CaCO3 at 37^o^C overnight. Samples of 1 ml were removed, placed on ice, then centrifuged at 16 000g for 1 min. Supernatants were removed and stored at −80°C before analysis by gas chromatography. Acetic acid, butyric acid, ethanol, acetone and butanol, were quantified using a Thermo Focus GC equipped with a 30 m TR-FFAP column (0.25 mm internal diameter) and a flame ionization detector as described by (Heap et al 2012). Exogenous uracil (20µg/ml) was added to the medium in the case of the *C. acetobutylicum* ATCC 824 *pyrE* mutant stain. Samples were extracted before injection by adding 500 μl of propyl propionate containing 50 mM valeric acid as the internal standard to the supernatant sample (500 μl), vortexing for 10 s and centrifuging for 1 min at 16 000g. The 300 μl organic phase was removed to a 2 ml sample vial containing a 300 μl deactivated glass insert. Samples of 1 μl were injected. Strains used were the *C. acetobutylicum* ATCC 824 wildtype (black filled bars), its *pyrE* mutant strain (grey filled bars) and the *pyrE* repaired strain (unfilled bars), in which the *pyrE* mutation was restored to the wildtype allele using the ACE correction vector pMTL-ME6.
